# MicroRNA-570 targets the HSP chaperone network, increases proteotoxic stress and inhibits mammary tumor cell migration

**DOI:** 10.1038/s41598-022-19533-6

**Published:** 2022-09-16

**Authors:** Yuka Okusha, Martin E. Guerrero-Gimenez, Benjamin J. Lang, Thiago J. Borges, Mary A. Stevenson, Andrew W. Truman, Stuart K. Calderwood

**Affiliations:** 1 Beth Israel Deaconess Medical Center, Harvard Medical School, Boston, MA 02215 USA; 2 JSPS Overseas Research Fellow, Tokyo, 102-0083 Japan; 3 Institute of Biochemistry and Biotechnology, School of Medicine, National University of Cuyo, 5500 Mendoza, Argentina; 4 Center for Transplantation Sciences, Department of Surgery, Massachusetts General Hospital, Harvard Medical School, Boston, MA 02129 USA; 5 Department of Biological Sciences, The University of North Carolina at Charlotte, Charlotte, NC 28223 USA

**Keywords:** Cancer, Cell biology

## Abstract

The dynamic network of chaperone interactions known as the chaperome contributes significantly to the proteotoxic cell response and the malignant phenotype. To bypass the inherent redundancy in the network, we have used a microRNA (mir) approach to target multiple members of the chaperome simultaneously. We identified a potent microRNA, miR-570 that could bind the 3′untranslated regions of multiple HSP mRNAs and inhibit HSP synthesis. Transfection of cells with this miR species reduced expression of multiple HSPs, inhibited the heat shock response and reduced tumor cell growth while acted additively in combination with cytotoxic drugs. As overexpression of miR-570 elicited tumor suppressive effects, we inferred that this miR could play a potential role in inhibiting tumorigenesis and cancer cell growth. In accordance with this hypothesis, we determined a significant role for miR-570 in regulating markers of mammary tumor progression, including cell motility and invasion. Our data provide a proof of the principle that the tumor chaperome can be targeted by microRNAs suggesting a potential therapeutic avenue towards cancer therapy.

## Introduction

Heat shock proteins (HSPs) are key stress proteins induced in cells exposed to proteotoxic insult and are critical for thermotolerance^1–3^. The canonical role played by HSPs is as molecular chaperones, folding nascent polypeptides, refolding denatured proteins and building multiprotein complexes^4,5^. The HSPs are also implicated in the pathophysiology of multiple diseases including cancer where elevated chaperone levels promote tumorigenesis^6,7^.

The major HSP families include HSP60, HSP70, HSP90 and TriC chaperones and the small HSPs^8,9^. Within these HSP families, HSP70 and HSP90α have become specialized to play crucial roles in cancer progression^10,11^. While these proteins retain their essential properties in maintaining proteostasis, their activities and specificities are dictated by helper co-chaperone proteins^12^. Of some significance in cancer, it has been shown that the HSP70 co-chaperone BAG3 can be targeted in mammary tumor growth^10^.

The versatile roles of HSPs in cell physiology are partially attributed to the formation of multiprotein chaperone /co-chaperone networks that can be very large^13–16^. Such chaperone networks appear to be highly dynamic and can be modified upon exposure to a variety of conditions, including heat shock, DNA damage, cell cycle progression and chaperone post-translational modifications^17–22^. In addition, under pathological conditions including cancer, these members of the proteome complex can bind together in tight association, resulting in structures known as epichaperomes that play key roles in tumorigenesis and can determine sensitivity to anticancer drugs such as Hsp90 inhibitors^23–25^. Targeting these networks may therefore suggest a novel approach to anticancer therapy.

We have investigated the potential use of a microRNA approach to simultaneously target multiple members of the cancer chaperome. MicroRNAs are short, single-stranded RNA species typically around 22 bp in length, found in cells of many types that can silence the expression of intracellular target mRNA typically by binding to 3′ regulatory sequences (3’ UTR) found in many mRNA species^26,27^*.* The most common approach in target prediction is based on miRNA seed sequences (nucleotides 2–7 of a miRNA) and their complementary canonical binding sites^28^. In seed sequences, 7–8 nt sites mediate the bulk of the repression for each miRNA and are the sites identified by the most effective target-prediction tools^28,29^. Nonetheless, the 6 nt sites that either match only the seed or are offset by one nucleotide in either the 5′ or 3′ direction can also mediate detectable repression^30,31^. In addition, the 5 nt sites have been suggested to regulate target genes^32,33^*.* The binding of microRNAs sequence to mRNA 3’UTR leads to the generation of dsRNA that can be detected within the cell, leading to a reduction in translation^34^. Considering the redundancy that exists in the chaperome network, we hypothesized that a microRNA targeting approach could enable simultaneous inactivation of multiple chaperone proteins and inactivation of tumorigenic complexes. To this end, we aimed to identify microRNA species that might target multiple members of the chaperome and thus act as potential cancer therapeutic agents.

## Results

### The cancer chaperone network and tumor progression

**Figure 1:**
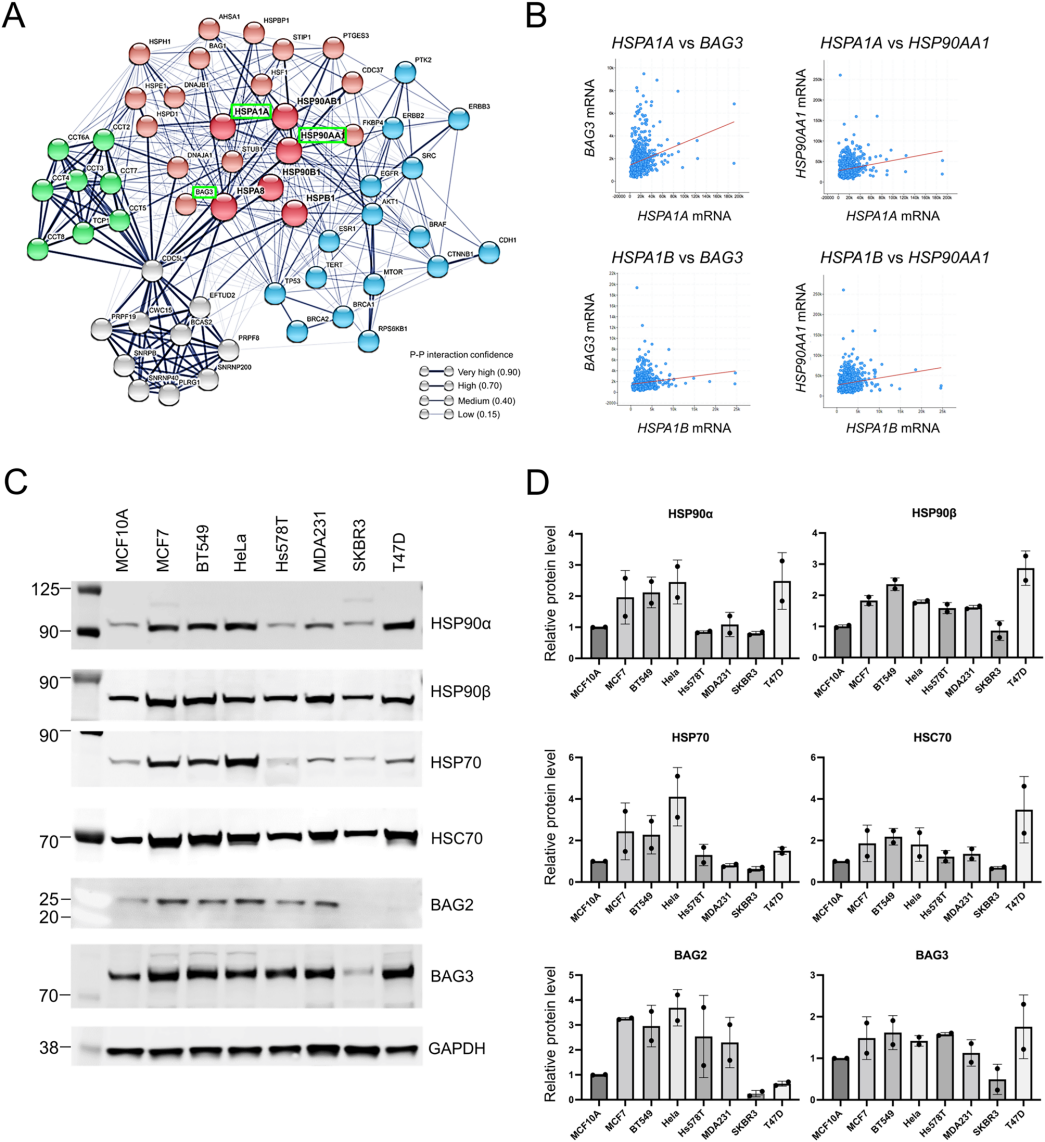
The interaction network of HSPs and co-chaperones in cancer progression. (**A**) Pattern of the gene interaction network of the HSPs, co-chaperone and cancer related genes. Red nodes: HSPs and co-chaperones, blue nodes: cancer-related genes, green nodes: TRiC complex genes, gray nodes: Prp19/CDC5L complex genes. The thickness of the border line indicates the strength of the experimental data supporting a protein–protein interaction. (**B**) Scatter plot analysis showing co-expression correlation between *HSPA1A* vs *BAG3*, *HSPA1A* vs *HSP90AA1*, *HSPA1B* vs *BAG3*, *HSPA1B* vs *HSP90AA1* in patient-derived breast tumor samples (960 cases). (**C**) Western blot analysis of HSP90α, HSP90β, HSP70, HSC70, BAG2, BAG3 and GAPDH in the normal MCF10A human mammary epithelial cell line, cultured human breast cancer cell lines and HeLa cervical adenocarcinoma cells. Original blots are presented in Supplementary Fig. S5. (**D**) Quantitative analyses of western blot for HSP90α, HSP90β, HSP70, HSC70, BAG2, BAG3. GAPDH was used as an internal control. Molecular weight markers are indicated in kilodalton. The data shows mean ± SD obtained in the biological duplicate assay and the individual data points were indicated in a circle.

To characterize functional HSP chaperone complex networks, we first analyzed *HSP* gene interactions using the STRING database. At the hub of our generated network were important tumor-promoting chaperones and co-chaperones such as HSP90, HSP70 and BAG3 (Fig. 1A). We therefore next examined the co-expression correlation between HSPs and co-chaperones including HSP90 family (*HSP90AA1*, *HSP90AB1*), HSP70 family (*HSPA1A*, *HSPA1B*, *HSPA8*) and BAG family (*BAG1*, *BAG2*, *BAG3*) among 960 breast tumor samples registered in the Cancer Genome Atlas (TCGA). There was significant co-expression among HSPs and co-chaperones (Table S1). Among these, we observed a marked co-expression significance between *HSPA1A* and *BAG3* (y = 0.02x + 1421.7, *p* = 1.83E−25), *HSPA1A* and *HSP90AA1* (y = 0.25x + 27,649.4, *p* = 1.12E−11), *HSPA1B* and *BAG3* (y = 0.1x + 1487.6, *p* = 4.76E−14), *HSPA1B* and *HSP90AA1* (y = 1.72x + 27,142.6, *p* = 1.15E−17) in the breast tumor cases (Fig. 1B). To further study the importance of the chaperome in cancer cell lines, we examined the expression level of HSPs and co-chaperones in human breast cancer cell lines and HeLa cells. Across the cell lines examined, HSPs and co-chaperone levels of MCF7, BT549, Hela and T47D were higher than those in MCF10A, normal mammary epithelial cells (Fig. 1C,D). The levels of HSPs were lower in SKBR3 than in MCF10A. These data suggested that the extent of the chaperone network maintains some relationship with cancer prognosis, suggesting that single protein inhibition may not be sufficient to attenuate this chaperome network. Of further significance, the relative expression patterns of HSP90α and HSP70 were found to follow a similar pattern, with levels observed in MCF7, BT549, HeLa and T47D higher than those of MCF10A (Fig. 1C,D). Data are in concordance with prior studies which suggest that chaperones are co-expressed in many breast cancers and that HSPs present distinctive gene expression patterns that are partially related to the different breast cancer subtypes and patients^6^.

### Identification of miRNAs targeting HSPs and co-chaperones

**Figure 2:**
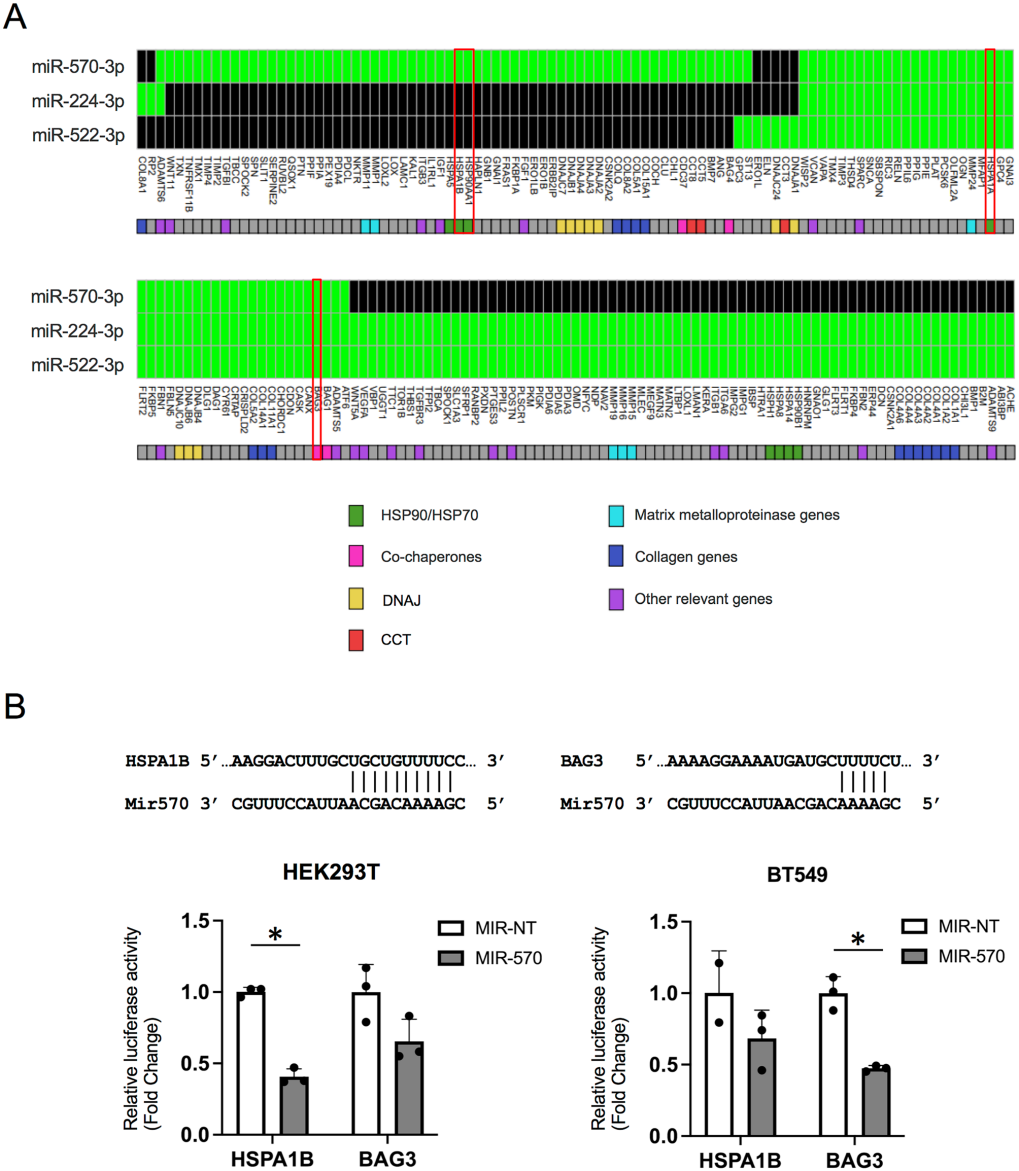
Determination of novel miRNAs targeting *HSPs* and *BAG3.* (**A**) Targeting genes of three candidate miRNAs in chaperome and ECM related genes. Green; the predicted positive target genes for each of the microRNA in the rows. Black; no predicted binding between the molecules studied. (**B**) Upper: Scheme of binding of miR-570 to *HSPA1B* and *BAG3* mRNA. The figure shows sequences from the 3′UTR regions of the targeting *HSPA1B* and *BAG3* genes and the microRNA 570 predicted to bind to them. Lower: the effect of miR-570 on reporters, such as pLightSwich_3UTR_HSPA1B and pLightSwich_3UTR_BAG3. Luminescence was measured for each condition to assess binding and effectiveness of miR-570. Representative images of two individual experiments with triplicates each. (n = 3; *p < 0.05, Student's t-test, error bars = SD).

To uncover candidate microRNAs targeting the tumor cell chaperone network, we utilized the *miRanda algorithm*^35^. From miRNAs potentially targeting HSPs and co-chaperones, we selected three candidates, miR-570, miR-224 and miR-522 (Fig. 2A). These miRNAs showed partial complemental sequences with 3′-UTR sequences in human *HSPA1A* and *BAG3* (Fig. 2, Fig. S1). Importantly, among three miRNAs, only miR-570 showed partial complemental with 3′-UTR sequences in not only human *HSPA1A* and *BAG3* but also *HSPA1B* and *HSP90AA1* (Fig. 2, highlighted in red, Fig. S1A), which are involved in cancer progression^10,11^. In addition, the HSP90 co-chaperone CDC37, a key protein in Hsp90 function in cancer^36^ was also potentially targeted by miR-570 (Table S2). We next examined potential targets of these three candidate miRNAs within the chaperome including the DNAJ and CCT (Chaperonin containing tailless complex polypeptide 1) genes and extracellular matrix (ECM) related genes including MMP (matrix metalloproteinase) genes and collagen genes. These three candidate miRNAs were predicted to also target collagen genes, MMP genes and chaperones such as DNAJ or CCT as well as HSPs and co-chaperones (Fig. 2A, Table S2). Our data suggested that although three candidate miRNAs could target the chaperone network and ECM related genes, miR-570 was different in potentially targeting each of *HSPA1A*, *HSPA1B*, *BAG3* and *HSP90AA1*, which are key chaperones and co-chaperones in the proteotoxic stress response. We therefore focused our following studies on determining the efficacy of miR-570 in targeting HSPs and its effect on intracellular chaperone function.

We next asked whether miR-570 could interact with the 3′UTRs of key members of the chaperome. We used a luciferase reporter assay in which the 3′-UTR potential miR-570-binding sites of *HSPA1B* and *BAG3* mRNAs are each fused to the luciferase gene. To determine the potential of miR-570 to interact with 3′UTRs, we used a construct that mimics miR-570, ds-miR-570 (mir-570). Co-transfection of miR-570 along with the expression plasmid containing the 3′-UTRs and luciferase reporter resulted in a decrease in luciferase activity levels for both *HSPA1B* and *BAG3* in HEK293T and BT549 cells (Fig. 2B), strongly suggesting that miR-570 targets the mRNAs through the 3′ regions and reduces expression of adjacent protein coding sequences.

### Native miR-570 gene region is methylated and expressed at low levels in breast tissues

**Figure 3:**
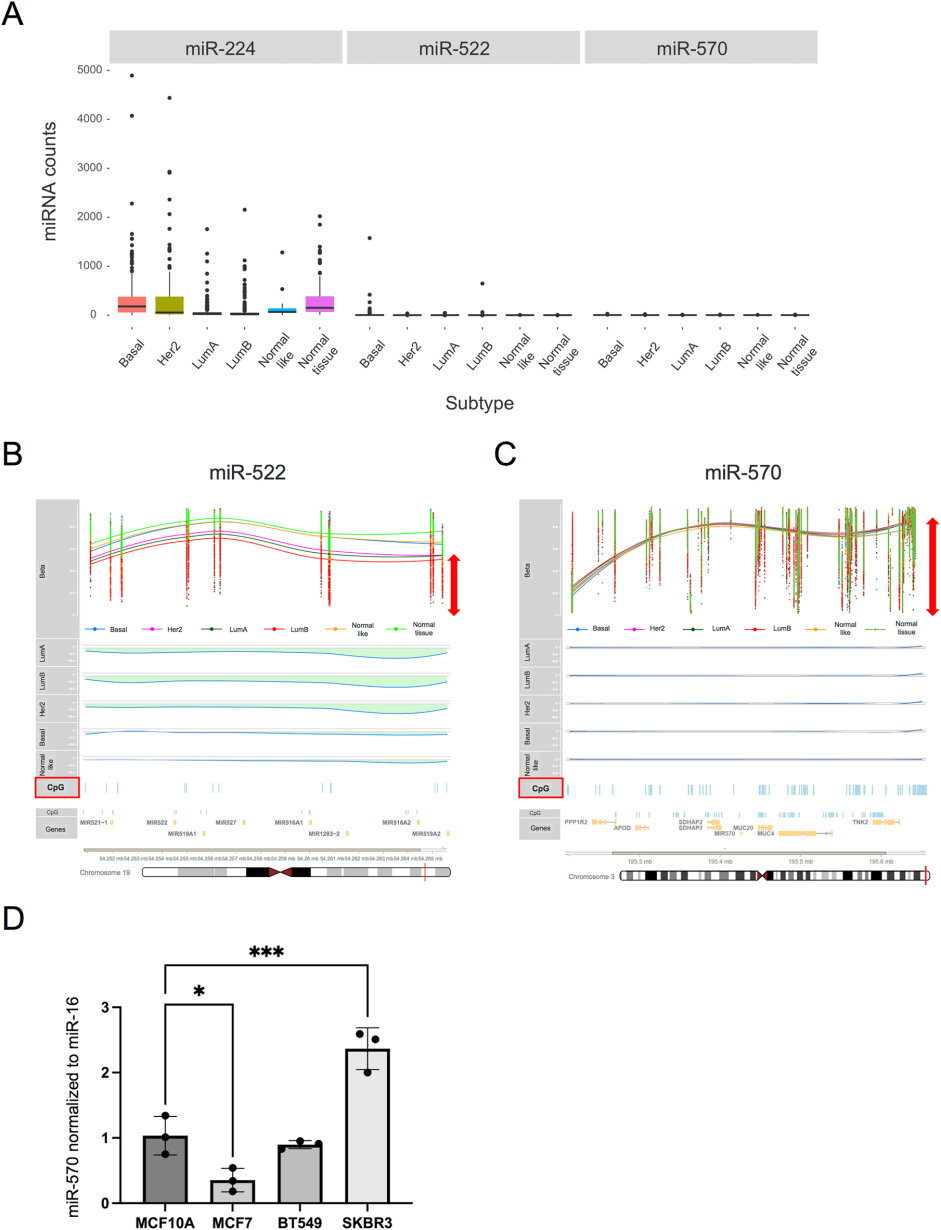
Gene expression and methylation status of three candidate miRNAs gene in breast tissue. (**A**) The total expression levels of the three miRNAs in the breast cancer cohort were evaluated by comparing their expression between the normal tissue and the different subtypes of tumor tissue for the candidate miRNAs. (**B**) Methylation status of the hsa-miR-522 gene in breast tumor samples. "Beta" lane shows the methylation values (Beta values) which range from 0 to 1 and are shown on the vertical axis (y-axis), while each column of dots represents a particular CpG site, located in relation to its chromosomal region (red line on the chromosome diagram at the bottom). Each point represents an individual sample and the methylation value measured for the corresponding CpG site. In the beta value lane, a smoothed regression curve was added to the scatter plot to show the methylation differences between mammary cancer subtypes and normal tissue. The lanes below show the *'bumps'* for each tumor subtype, representing the levels of methylation in each region as a whole. (**C**) Methylation status of the hsa-miR-570 gene in breast tumor samples. (**D**) miR-570 expression in MCF10A and breast cancer cell lines was evaluated through real-time PCR. Representative image of three individual experiments with three replicates (n = 3; *p < 0.05, ***p < 0.001, one-way ANOVA followed by post hoc Turkey’s test, error bars = SD).

To explore the relationship between miR-570 and breast cancer, we next investigated the normal human tissue miR-570 methylation and expression in different subtypes of breast tumors using the TCGA cohort. Surprisingly, the expression of miR-570 was not detected in either normal or tumor tissues in the database, while both miR-224 and miR-522 were observed in the analyzed breast tumor samples (Fig. 3A). Notably, miR-224 expression in breast tumor samples was higher than levels of other miRNAs. Previously, reports have shown that *miR-124a* and *miR-127* were targets for DNA methylation and thus became silenced in cancer cells, prompting us to screen for potentially tumor-suppressive miRNAs not expressed in tumor cells^37^. Our rationale was that silenced microRNAs were likely to be functional in a tumor-suppressive mode, as was observed in the seminal studies with the let-7 microRNA^38,39^. We therefore compared the CpG islands and methylation states of miR-522 (Fig. 3B) and miR-570 (Fig. 3C). The methylation level of miR-570 in breast cancer cases was higher than that of miR-522 (mean methylation value of tumor samples for mir-522, 0.62 and for mir-570, 0.7, Fig. 3C, red arrow) and the methylation levels of miR-522 tended to decrease in tumor tissues compared with non-tumor samples. miR-570 was however detected in mammary tissue culture cells although no clear pattern was detected. Expression of mir-570 in MCF7 was significantly lower than that of normal mammary epithelial cells MCF10A but this was not the case of SKBR3 (Fig. 3D). Interestingly, the miR-570 expression patterns were correlative with HSPs levels (Fig. 1C,D). These data suggested that miR-570 could be relatively silenced in both normal tissue and breast cancer cases and this status is possibly associated with increased methylation status. However future studies will be required to determine the regulation of miR-570 in mammary cancer and the role of methylation in normal and pathological states.

### Characterization of HSPs and BAG3 as novel direct targets of miR-570

**Figure 4:**
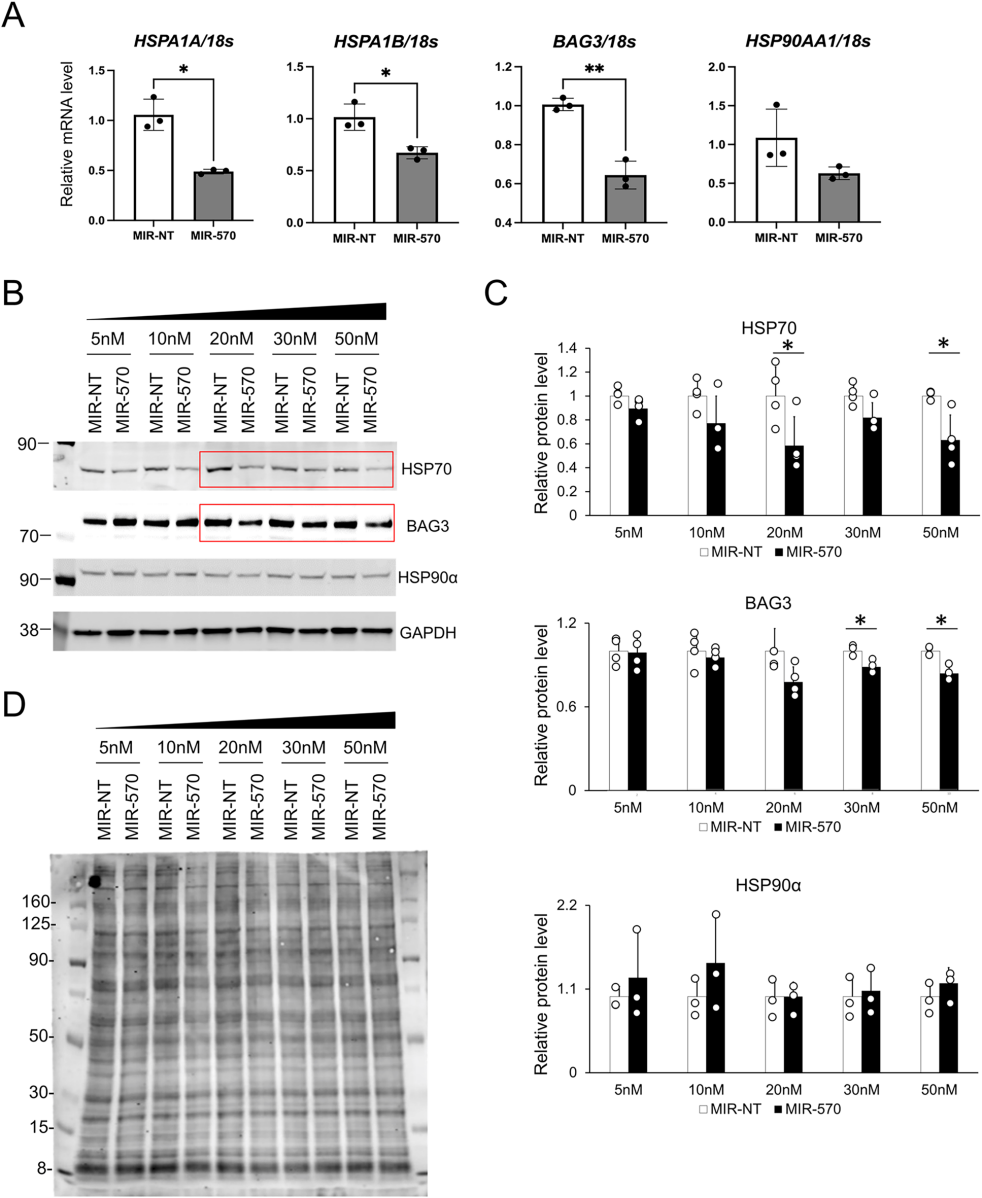
Characterization of HSP70, BAG3 and HSP90α as novel direct targets of miR-570. (**A**) Real-time qPCR analysis of *HSPA1A*, *HSPA1B*, *BAG3*, *HSP90AA1* in HEK293T cells 72 h after transfection with 25 nM (for *BAG3*) or 50 nM (for *HSPA1A*, *HSPA1B* and *HSP90AA1*) of dsRNA mimicking miR-570 (MIR-570) or control non-specific miRNA (MIR-NT). Representative image of two individual experiments with three replicates (n = 3; *p < 0.05, Student's t-test, error bars = SD). (**B**) Western blot analysis of HSP70, BAG3, HSP90α and GAPDH in HEK293T cells 72 h after transfection with a range of concentrations, 5 nM to 50 nM, of miR-570 or miR-NT. Molecular weight markers are indicated in kilodalton. Original blots are presented in Supplementary Fig. S6. (**C**) Quantitative analyses of western blot for HSP70, BAG3 and HSP90α of three or four individual experiments. Individual data points are indicated by open circles. (*p < 0.05, Student's t-test, error bars = SD). GAPDH was used as an internal control. (**D**) Total protein staining in HEK293T cells 72 h after transfection with 5 nM to 50 nM of miR-570 or control non-targeting miRNA of three individual experiments. The amount of protein sample was loaded 5 μg per each lane. Molecular weight markers are indicated in kilodalton.

To determine the potency of miR-570 in targeting gene expression, we used the construct described above that mimics miR-570, ds-miR-570 (MIR-570) or ds-miR-NT (MIR-NT). Transfection of HEK293T cells with ds-miR-570 resulted in a significant reduction of *HSPA1A, HSPA1B* and *BAG3* mRNAs 72 h post-transfection (Fig. 4A). The expression level of *HSP90AA1* also tended to decrease with ds-miR-570 transfection. Moreover, the expression of *HSPA5* and *CDC37* mRNA were also significantly reduced by miR-570 transfection as determined by miRanda (Fig. S2, Table S2). CDC37 is a major co-chaperone and is essential for permitting Hsp90 to chaperone protein kinases^36^. It can additionally perform stand-alone chaperone functions and is thus a component of the chaperome. To confirm a corresponding effect of ds-miR-570 on HSP70, BAG3 and HSP90α protein levels, lysates from cells transfected with ds-miR-570 were subjected to immunoblot analysis for detection of HSP70, BAG3 and HSP90α. Although HSP70 and BAG3 protein levels were impacted relative to the control treatment, HSP90α was not markedly altered (Fig. 4B,C). As the observed decreases in chaperone abundance could potentially be attributed to a decline in general translation, we also analyzed the overall patterns of protein expression by SDS-PAGE and total protein staining. These experiments did not, however, indicate major overall declines in the expression pattern with ds-miR-570 treatment (Fig. 4D). Conversely, the products of chaperone genes not predicted to be miR-570 targets, such as HSP90β, HSC70 and BAG2 were not significantly changed by miR-570 transection (Fig. S3).

### Role of miR-570 in cellular resistance to proteotoxic stress

**Figure 5:**
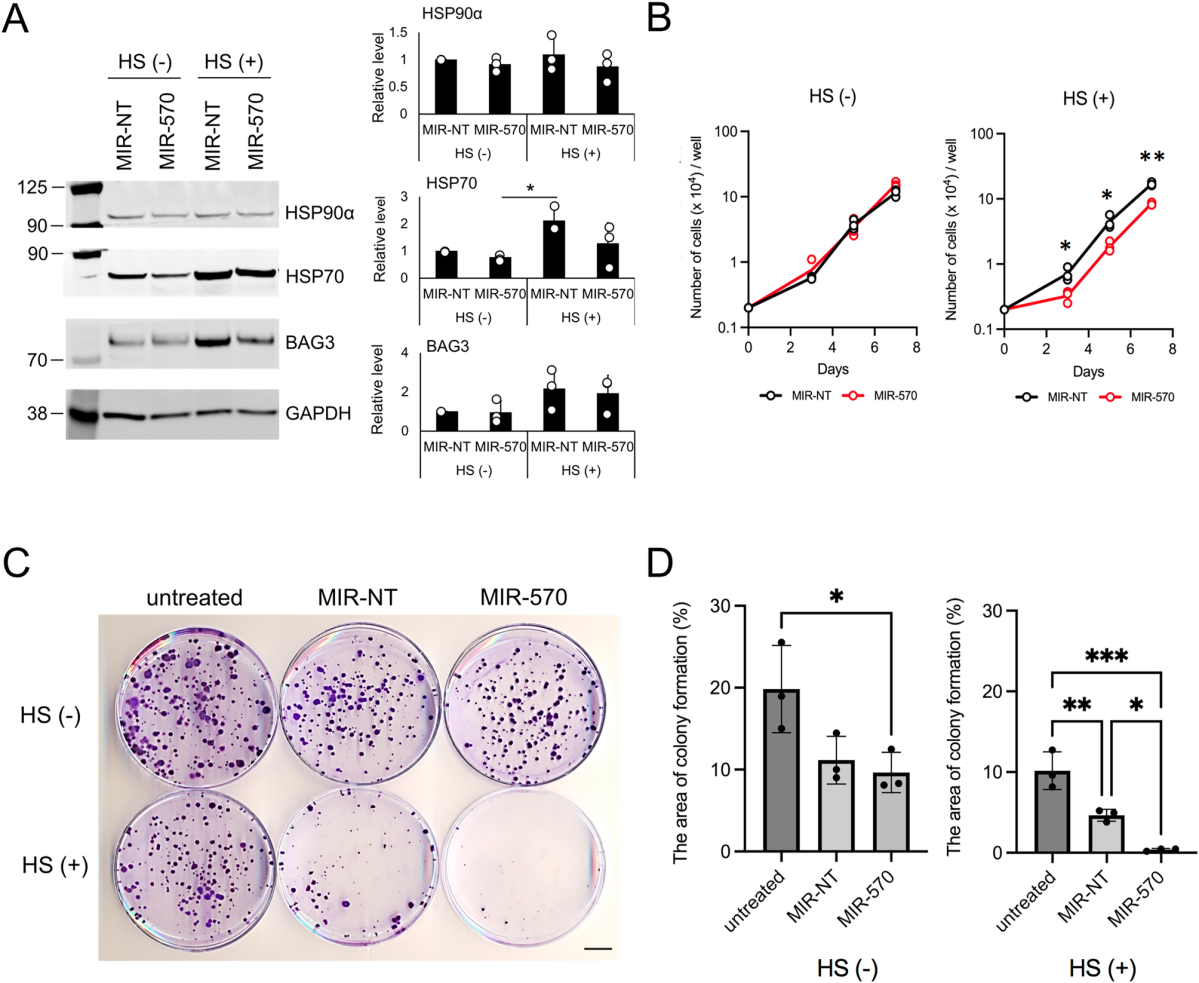
The role of miR-570 in cellular resistance to proteotoxic stress. (**A**) Western blot analysis of HSP90α, HSP70, BAG3 and GAPDH in HEK293T cells 72 h after transfection with miR-570 (50 nM) or MIR-NT (50 nM) and heat shock treatment. HS (−): untreated, HS (+): heat shock treatment. Quantitative analyses of western blot for HSP90α, HSP70 and BAG3 of three individual experiments and the individual data points are indicated by the open circles (n = 3; *p < 0.05, one-way ANOVA followed by post hoc Turkey’s test, error bars = SD). Levels of GAPDH expression was used as loading control. Molecular weight markers are indicated in kilodalton. Original blots are presented in Supplementary Fig. S7. (**B**) Growth curves of control non-targeting miRNA and mir-570 cells. Representative image of three individual experiments with three replicates (n = 3; *p < 0.05, **p < 0.01, Student's t-test, error bars = SD). (**C**) Representative image of colony formation in untreated or heat shock treated HEK293T cells, control non-targeting miRNA and mir-570 cells stained with crystal violet. Scale bar, 1 cm. (**D**) Quantitative analyses of colony formation numbers. Percentage of colonies formed was calculated using ImageJ. Representative image of three individual experiments with three replicates (n = 3; *p < 0.05, **p < 0.01, ***p < 0.001, one-way ANOVA followed by post hoc Turkey’s test, error bars = SD).

Activation of the heat shock response leads to the synthesis of HSPs that regulate resistance to an array of stresses, including heat itself and lead to thermotolerance^1^. We therefore investigated the role of miR-570 in this response to proteotoxic stress in HEK293T cells. As temperature sensitivity is tightly regulated by upregulated HSP expression in many cells, we also examined the effects of ds-miR-570 transfection on the expression of HSP90α, HSP70 and BAG3 after heat shock. Heat shock led to abundant increase in the expression of HSP90α, HSP70 and BAG3 as determined by western blotting (Fig. S4A). miR-570 transfection decreased the expression of each of the proteins induced by heat shock (Fig. 5A, Fig. S4B,C). We next examined the ability of miR-570 to regulate the sensitivity to heat shock using cell proliferation and clonogenic cell survival assays. In the proliferation experiments, we observed minimal difference between miR-NT and miR-570 transfection in controls with no exposure to heat shock, whereas exposure to heat shock at 43 °C significantly decreased the cell proliferation in miR-570 transfectants compared to miR-NT (Fig. 5B). For the colony formation cell survival assay, the cell strain used in these studies was approximately 20% clonogenic prior to stress (Fig. 5C,D). Colony formation was decreased in samples transfected with miR-570 compared to the untreated group and there was no detectable difference in clonogenicity among non-heat shocked cells transfected with either miR-NT or miR-570. However, exposure to heat shock at 43 °C led to a profound decrease in colony formation potential in both miR-NT and miR-570 treated conditions (Fig. 5C,D). These decreases of colony formation potential in heat-shocked cells relate to the effects of both transfection and heat shock stress. However, treatment with miR-570 almost completely eradicated the heat shock response in these cells. These data confirm that HSP levels are closely related to cell survival and thermotolerance during proteotoxic stress imposed by heat shock and show that homeostasis by the HSP network was antagonized by miR-570 mimic transfection.

### miR-570 transfection reduces HSP levels in mammary cancer cells and sensitizes cells to Hsp90 inhibitory drugs

**Figure 6:**
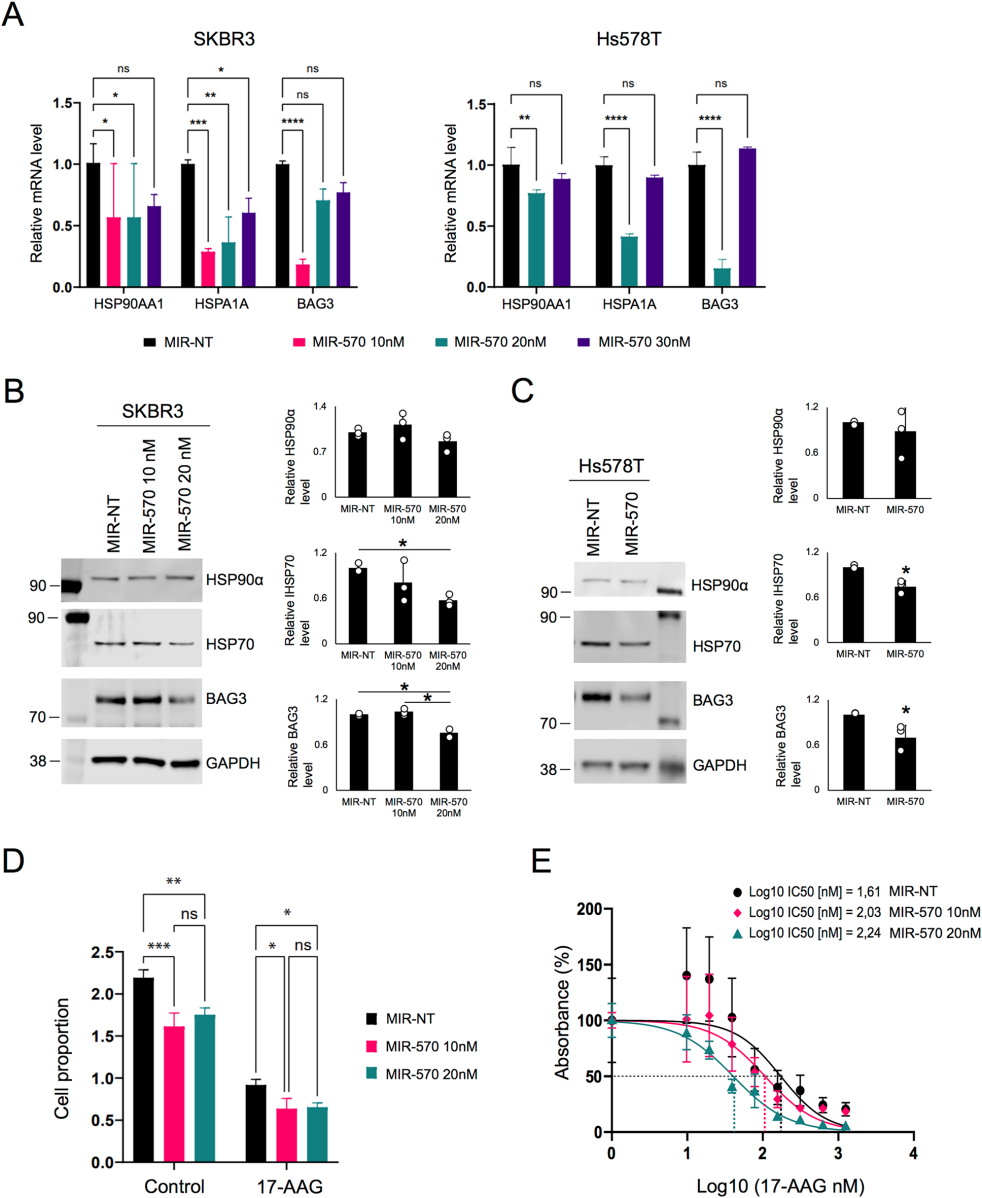
The suppressive effects of miR-570 on HSP and BAG3 expression in mammary cancer cells. (**A**) Expression of chaperones *HSP90AA1, HSPA1A* and co-chaperone *BAG3* assayed by RT-qPCR after treatment with mir-570 in breast cancer cell lines. The value expressed is the relative quantification to the *TMEM11* house-keeping gene for each condition. (n = 3; ns = no significance, *p < 0.05, **p < 0.01, ***p < 0.001, ****p < 0.0001, error bars = SEM). (**B**) Western blot analysis of HSP90α, HSP70, BAG3 and GAPDH protein expression in SKBR3 cells 72 h after transfection with 10 nM or 20 nM of miR-570 or MIR-NT. Quantitative analyses of western blot for HSP90α, HSP70 and BAG3 of three individual experiments and the individual data points were indicated in a circle. (n = 3; *p < 0.05, one-way ANOVA followed by post hoc Turkey’s test, error bars = SD). GAPDH was used as an internal control. Molecular weight markers are indicated in kilodalton. Original blots are presented in Supplementary Fig. S8. (**C**) Western blot analysis of HSP90α, HSP70, BAG3 and GAPDH protein expression in Hs578T cells 72 h after transfection with 50 nM of miR-570 or MIR-NT. Quantitative analyses of western blot for HSP90α, HSP70 and BAG3 of three individual experiments and the individual data points were indicated in a circle. (n = 3; *p < 0.05, Student's t-test, error bars = SD). GAPDH was used as an internal control. Molecular weight markers are indicated in kilodalton. Original blots are presented in Supplementary Fig. S9. (**D**) Assay of cell proliferation with and without the Hsp90 inhibitor 17-AAG in cells treated with miR-570. After treatment with miR-570 or non-silent miRNA (MIR-NT), the proliferation of SKBR3 cells was evaluated with and without treatment with 17-AAG. Cells were counted by brightfield imaging cytometry at time 0 and after 72 h. The value shown is the cell quantification relative to time 0 for each condition. (n = 3; ns = no significance, *p < 0.05, **p < 0.01, ***p < 0.001, error bars = SEM). (**E**) Dose–response curves with IC50 values for 17-AAG in respect to treatment to miR-570 and MIR-NT. The results are expressed as percentage of absorbance measurement of MTT assay. The calculated regression curve is indicated as a line. (n = 3, error bars = SEM).

To examine whether miR-570 impacts the expression of its predicted targets in tumorigenic breast cancer cell lines, we performed a similar experiment to the one in Fig. 4, using SKBR3 and Hs578T mammary cancer cells instead. We observed that mRNA levels of HSP90AA1, HSPA1A and BAG3 were significantly reduced 72 h after transfection of the dsRNA mimicking miR-570 (Fig. 6A). Expression of the HSP mRNAs was uniformly reduced by transfection with 10 and 20 nM miR-570 although contrasting results were observed above these levels perhaps indicating off-target effects at these higher concentrations. Levels of HSP70 and BAG3 proteins were also reduced after overexpression of ds-miR-570 in these breast cancer cell lines (Fig. 6B,C). We next assayed cell proliferation with and without the Hsp90 inhibitor 17-AAG in SKBR3 cells treated with miR-570. After treatment with miR-570 or miR-NT, the proliferation of only miR-570 transfected cells decreased. After additional treatment with 17-AAG, cell proliferation decreased further than with miR-570 alone. However, when both 17-AAG and miR-570 were combined proliferation decreased more than with each agent independently (Fig. 6D). In the MTT assay, the IC50 of 17-AAG was reduced in a dose-dependent manner with increasing miR-570 levels (Fig. 6E). These data confirmed that HSP levels are closely related to tumor cell survival during proteotoxic stress imposed by an Hsp90 inhibitor and that homeostasis was antagonized by miR-570 mimic transfection.

### MiR-570 mimicking constructs impact tumor cell growth and migration

**Figure 7:**
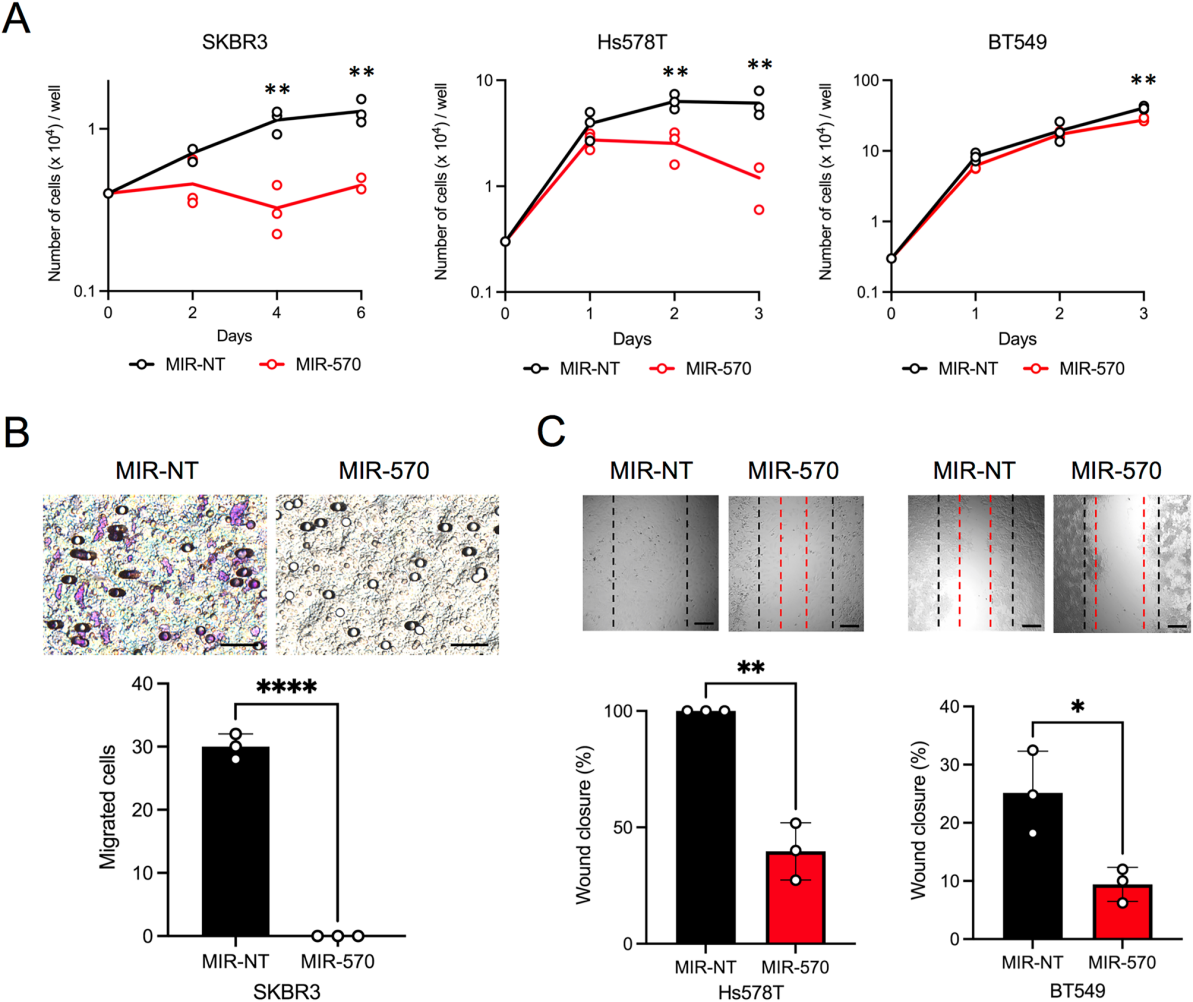
Effects of miR-570 on indices of mammary cell growth and migration. (**A**) Growth curves of control non-targeting miRNA or miR-570 transfected SKBR3, Hs578T and BT549 cells. Representative image of three individual experiments with three replicates (n = 3; **p < 0.01, Student's t-test, error bars = SD). (**B**) Upper: representative images of migrated cells that have migrated through filters and migration activities of SKBR3 cells treated with control non-targeting miRNA or miR-570. Scale bars, 20 μm. Lower image shows quantitation of numbers of cells migrating through the filter (means ± SD). Representative image of three individual experiments with three replicates (n = 3; ****p < 0.0001, Student's t-test, error bars = SD). (**C**) Upper: representative cell culture images at 24 h after the wounding. Dashed black line: initial wounds. Dashed red lines: closed wounds at 24 h. Scale bars, 100 μm. Lower images show the relative rates of the wound closure. Representative image of three individual experiments with three replicates (n = 3; *p < 0.05, **p < 0.01, Student's t-test, error bars = SD).

In addition to protecting cells from proteotoxic stresses, HSPs have been shown to play roles in tumorigenesis^3,10,40^. To investigate the potential impact of miR-570 on indices of tumor progression, we performed proliferation, cell migration and cancer cell monolayer wound healing assays on miR-570-transfected mammary carcinoma cells. In the proliferation assay, miR-570 transfected cells grew more slowly compared to controls in each of the SKBR3, Hs578T and BT549 cell lines analyzed (Fig. 7A). The rate of cellular migration was also markedly inhibited by ds-miR-570 transfection in SKBR3 cells compared to cells transfected with the control miRNA (Fig. 7B). In addition, the cell monolayer wound closure rate, another index of cell migration, was decreased after miR-570 transfection in Hs578T and BT549 cells (Fig. 7C). Overall, these data suggested that miR-570 function could suppress some of the tumorigenic properties of breast cancer cells, with marked effects on ability to migrate, a rate limiting property in metastasis^41^.

### Direct HSP70/BAG3 siRNA knockdown impact tumor growth and migration

**Figure 8:**
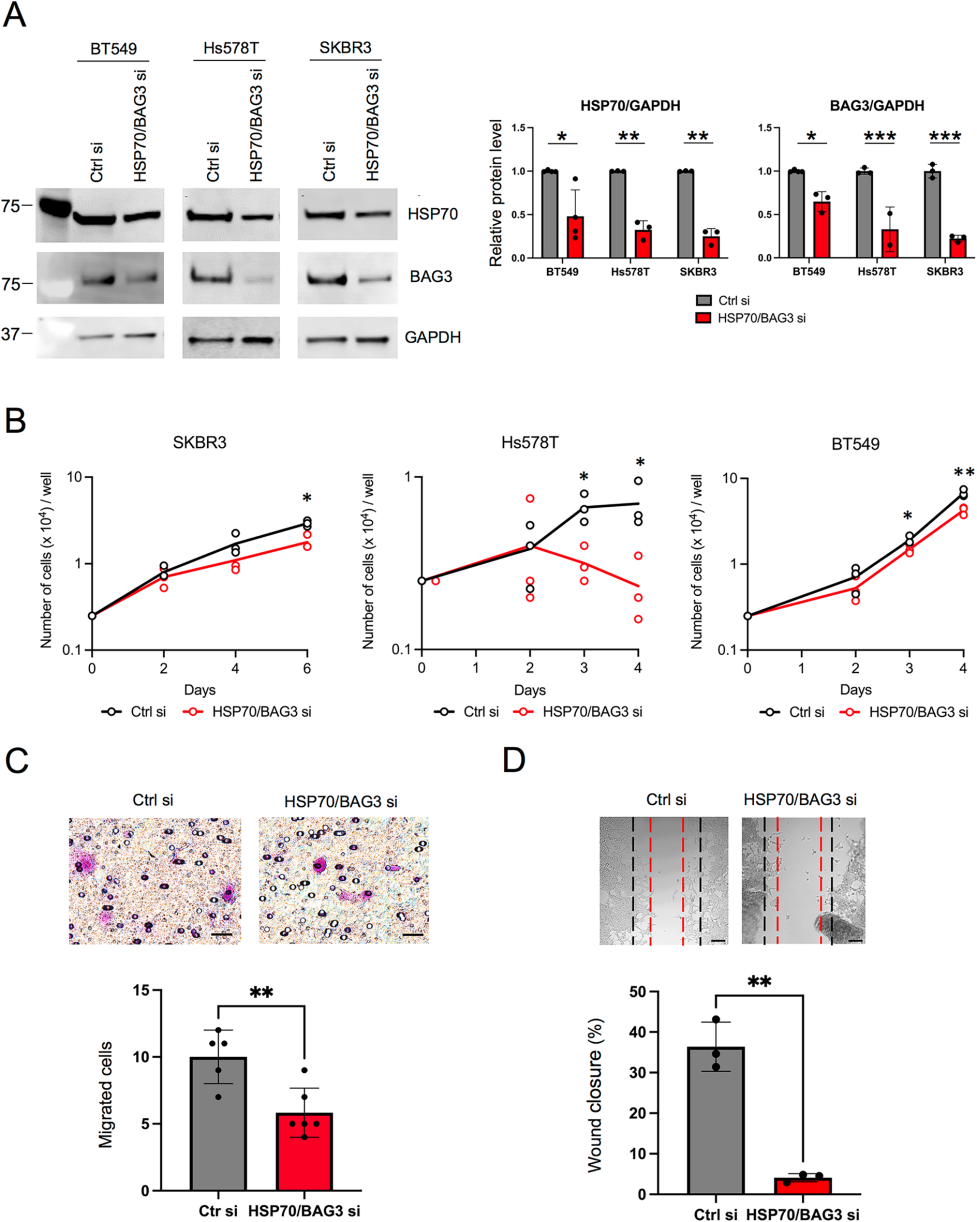
Direct HSP70/BAG3 siRNA knockdown impact tumor growth and migration. (**A**) Western blot analysis of HSP70, BAG3 and GAPDH protein expression in breast cancer cell line 72 h after transfection with 20 nM of HSP70/BAG3 siRNA or non-targeting siRNA. Quantitative analyses of western blot for HSP70 and BAG3 of three individual experiments and the individual data points were indicated in a circle. (n = 3; *p < 0.05, **p < 0.01, ***p < 0.001, Student's t-test, error bars = SD). GAPDH was used as an internal control. Molecular weight markers are indicated in kilodalton. Original blots are presented in Supplementary Fig. S10. (**B**) Growth curves of control siRNA or HSP70/BAG3 siRNA transfected SKBR3, Hs578T and BT549 cells. Representative image of three individual experiments with three replicates (n = 3; *p < 0.05, **p < 0.01, Student's t-test, error bars = SD). (**C**) Upper: representative images of migrated cells that have migrated through filters and migration activities of SKBR3 cells treated with control non-targeting siRNA or HSP70/BAG3 siRNA. Scale bars, 20 μm. Lower image shows quantitation of numbers of cells migrating through the filter (means  ± SD). Representative image of three individual experiments with three replicates (n = 3; **p < 0.01, Student's t-test, error bars = SD). (**D**) Upper: representative cell culture images at 24 h after the wounding. Dashed black line: initial wounds. Dashed red line: closed wounds at 24 h. Scale bars, 100 μm. Lower images show the relative rates of the wound closure. Representative image of three individual experiments with three replicates (n = 3; **p < 0.01, Student's t-test, error bars = SD).

As miR-570 appears to be somewhat pleiotropic as regards molecular targets, we investigated whether its effects could be replicated using siRNA constructs specific for HSP70 and BAG3, chaperones that seem to be central components of the chaperome (Fig. 8A). Indeed, HSP70 and BAG3 siRNA transfected cells grew more slowly compared to controls in each of the SKBR3, Hs578T and BT549 cell lines as had been observed with miR-570 (Fig. 8B). The rate of cellular migration was also markedly inhibited by HSP70 and BAG3 siRNA transfection in SKBR3 cells compared to cells transfected with the control siRNA (Fig. 8C). In addition, the cell monolayer wound closure rate was decreased by HSP70 and BAG3 siRNA transfection in BT549 cells (Fig. 8D). The potency of the siRNA combination was similar in magnitude to that of miR-570, confirming the potential direct targeting of the chaperome by the microRNA approach. These data are therefore consistent with Fig. 7 suggesting that HSP70 and BAG3 may be important targets of miR-570 in its effects on cancer cell growth and migration, and can these molecules be inactivated by introduction of a miR-570 mimicking agent.

## Discussion

Our data indicate that microRNA can regulate the intracellular chaperone network and thus play roles in regulating the proteotoxic stress response and some aspects of cancer biology (Figs. 1, 7). One species, in particular, miR-570 appeared able to target multiple HSP mRNAs and was selected as an agent potentially able to antagonize the functions of the chaperome (Fig. 2, Suppl Fig. S1). In addition, this species appeared to be quantitatively silenced in breast cancers sub-types in human databases, potentially through a mechanism potentially involving methylation (Figs. 1, 2, 3). A previous report had shown miR-570 to inhibit cell proliferation^42^, while the current report shows for the first time that miR-570 targets HSP chaperome network. Our data provide a proof-of-the principle that the chaperome could be targeted by microRNA and opens the door to a potential role of miRNAs in the stress response and the tissue functions of molecular chaperones.

We have now shown the canonical site types including 7 nt site, 6 nt site and offset 6 nt site between miR-570 and HSPs/BAG3 (Fig. 2B, Fig. S1). The binding site of BAG3 and miR-570 was a 5 mer, which was not a canonical site type, however, co-transfection of miR-570 along with the expression plasmid containing the 3′-UTRs and luciferase reporter resulted in a decrease in luciferase activity levels for BAG3 in BT549 cells (Fig. 2B). Indeed, both mRNA and protein levels of BAG3 were decreased by miR-570 transfection (Fig. 4). Consistent with the previous reports^32,33^, the 5 nt sites might also play an essential role for miRNA-target binding.

In these studies, we have also investigated the potential of reintroducing the gene product in the form of double-stranded oligonucleotide miR-570-3p into cells by transfection. Feasibility studies in HEK293T cells indicated a versatile inhibition of HSP70, HSP90α and BAG3 mRNA, key members of the chaperome (Fig. 4). Reductions in HSP mRNA and protein were by no means complete, and HSP70 and BAG3 protein levels in particular were significantly reduced only at the higher concentration of miR-570. The outcome of exposure to miR-570 partially depends on the stability of individual proteins. However, partial inhibitory effects on multiple members of the chaperome appears to have cooperative effects on the functioning of the chaperone network. Cells expressing miR-570 seemed to lose their capacity to mount the heat shock response, indicating profound inhibition of the homeostatic effects of HSPs and the chaperome in thermotolerance (Fig. 5). The significance of this microRNA species in breast cancer development was suggested by studies in mammary cancer cell lines which indicated reduced growth and loss of cell motility, important properties in transformation and tumor progression, after chaperone targeting (Figs. 6, 7, 8). HSPs have previously been found to play key roles in cancer and targeting them genetically or with small molecule inhibitors can reduce malignancy^40,43,44^. However, understanding of the mechanisms underlying molecular chaperone function in cancer is still in its infancy^45^. It will be apparent that the effects of each of the the microRNAs discussed here are somewhat pleiotropic, targeting MMP genes, co-chaperones and CCT chaperones (Fig. 2, Table S2) and molecules in addition to HSP70, HSP90α and BAG3 could be involved in the effects observed. An RNA-seq could be useful to see other important targets to elicit the tumor suppressive effects. We were however able to reproduce most of the effects of miR-570 with siRNA specifically targeting HSP70 and BAG3, indicating a key contribution of these members of the chaperome to the anticancer effects of miR-570 (Fig. 8).

The future use of miR-570 mimics in vivo and in a clinical setting would require an effective delivery system^46^. One possible approach that has been tested in several studies would be to enclose the therapeutic microRNA molecule in liposomal vectors before introduction into a host, and this approach has seen some success, although much of the compound would be delivered to non-tumor sites in this approach^47^. However, this methodology has the potential for specific tumor targeting by enclosing tumor binding / targeting ligands into the liposomes^48^.

In conclusion, we have shown the feasibility of targeting tumor cell chaperone networks with microRNA mimicking constructs and shown that the proteotoxic cell response, malignant cell growth and the cancer cell phenotype can thus be inhibited.

## Materials and methods

All the procedures were performed in accordance with the relevant guidelines and regulations.

### Bioinformatic analyses

The gene interaction network was analyzed using the STRING database (https://string-db.org) of the Swiss Institute of Bioinformatics and the European Molecular Biology Laboratory (EMBL)^49^. To construct the network, the web application was seeded with the chaperone and cancer genes of interest and it was configured to show interactions obtained from protein–protein interactions experiments and curated databases with a minimum interaction score of 0.15 and a second shell of interactors of up to 5 genes. After the network was obtained, proteins were reordered manually to show the distinct gene hubs depicted in Fig. 1A.

The miRNA species predicted to bind to elements of the chaperome were searched through 14 different miRNA databases using the 'multiMiR' package for R^50^. miRNAs can bind to a large number of genes at their 3'UTR site, thus, we filtered the miRNAs that were computationally predicted to bind HSP90AA1, HSPA1A, HSPA1B and BAG3 3′UTR sites. To further evaluate the binding context to the miRNA-Gen pair we used the “miRanda” algorithm^35^.

The “TCGA assembler” package v.1.0.3^51^ was used to programmatically download the transcriptomic and methylomic dataset from the mammary adenocarcinoma databases of TCGA (https://portal.gdc.cancer.gov/, accessed May 2015). Regarding the transcriptomic data, the gene expression levels of standardized miRNAs (normalized) of 1,078 tumor samples were obtained from the RNA-Seq data available for miRNAs (RNASeqV2). Tumors were classified into their different intrinsic molecular subtypes according to the PAM50 method^52^ using the “Bioclassifier” package (https://genome.unc.edu/pubsup/breastGEO/PAM50.zip accessed on October 2016).

To evaluate the methylation of the genomic regions close to the different candidate miRNAs, level 3 data of the methylation profiles of 842 samples were downloaded directly from the TCGA website (https://portal.gdc.cancer.gov/). In particular, the samples were analyzed using the platform Infinium HumanMethylation450K BeadChip from Illumina (JHU-USC-HumanMethylation450k), once the processed beta values ​were downloaded, the CpG sites in the genomic surroundings corresponding to the miRNAs of interest filtered using the probes annotations provided by the chip manufacturer. In order to determine if the miRNAs genomic regions show altered DNA methylation profiles in breast cancer, we decided to perform a comparison of methylation status between breast cancer subtypes and normal tissue. In order to identify differentially methylated loci in HSPs, the bumphunter algorithm^53^ was used using the 'Minfi' R package^54^. Bumphunter provides a robust method for detecting differentially methylated loci, as opposed to analyzing individual CpG sites, by performing probe-level regression and smoothing the coefficient of interest within clusters to identify "bumps" across the genome. For further information about biospecimen collection, processing, quality control and biomarker assessment^55^ or TCGA website (http://cancergenome.nih.gov). The co-expression correlation dataset of breast tumor (960 samples) from TCGA was analyzed using cBioportal^56^.

### Cell lines

Human embryonic kidney cell line HEK293T, human non-tumorigenic epithelial cell line MCF10A and human breast cancer cell lines Hs578T, MDA-MB231 and SKBR3 and HeLa cell line were obtained and cultured as recommended by American Type Culture Collection (ATCC, Gaithersburg, MD, USA). MCF7, BT549 and T47D were provided from Alex Toker’s lab (BIDMC, USA). HEK293T and HeLa were maintained in Dulbecco’s modified Eagle’s medium (DMEM, Gibco, Carlsbad, CA, USA) with 10% fetal bovine serum (FBS, Gibco, Carlsbad, CA, USA). MCF10A was maintained in F12 medium (DMEM-F12, Gibco, Carlsbad, CA, USA) supplemented with 5% horse serum, hydrocortisone (Invitrogen, Carlsbad, CA, USA), insulin (0.01 mg/mL, Sigma-Aldrich, St. Louis, MO, USA), epidermal growth factor (20 ng/mL, NJ, USA), hydrocortisone (0.5 mg/mL, Stemcell technology, Canada), cholera toxin (100 ng/mL) and penicillin–streptomycin (100 μg/mL each, Invitrogen, Carlsbad, CA, USA). MCF7 and Hs578T were maintained in DMEM supplemented with 10% FBS and human insulin (0.01 mg/mL, Sigma-Aldrich, St. Louis, MO, USA). BT549 and T47D were maintained in Roswell Park Memorial Institute 1640 (RPMI1640, Gibco, Carlsbad, CA, USA) with 10% FBS. MDA-MB231 was maintained in Leibovitz's L15 (ATCC, Gaithersburg, MD, USA) supplemented with 10% FBS. SKBR3 was maintained in McCoy's 5a Medium (ATCC, Gaithersburg, MD, USA) with 10% FBS.

### Luciferase assay

Luciferase reporter assay were performed using the LightSwitch Luciferase Assay System (Switchgear genomics, CA, USA) according to the manufacture’s instructions. Cells were plated into 96-well plates, triplicates for each condition. Twenty-four hours after seeding, the cells were co-transfection with the pLightSwich plasmid, which is used for internal normalization and each constructs containing the sequence of *HSPA1B* or *BAG3* mRNA 3′-UTR. Luciferase activity was measured with the LightSwitch Luciferase Assay System (Switchgear genomics, CA, USA).

### Analysis of miR-570 levels

To analyze the miR-570 level, real-time RT-PCR was conducted using TaqMan MicroRNA Assays (Applied Biosystems, CA, USA) and a Universal PCR Master Mix II (Applied Biosystems), according to the manufacturer’s instructions. The cycling conditions were done according to the manufacturer’s instructions. The primers and probes were defined as mir-570 (Assay ID: 002347). hsa-mir-16 (Assay ID: 000391; UAGCAGCACGUAAAUAUUGGCG) level was used as an internal control.

### Transfection with synthetic miRNAs and small interfering RNAs (siRNAs)

HEK293T, SKBR3, Hs578T and BT549 cells (1 × 10^5^ cells) were seeded and grown in 12-well plate. The cells were transiently transfected with 5 nM to 50 nM of Vana miR-570-3p mimic (MIR-570) or control non-targeting miRNA (MIR-NT, Ambion, Life technologies, Waltham, MA, USA), using Lipofectamine RNAiMAX Reagent (Invitrogen, Carlsbad, CA, USA) following the manufacturer's protocol. mirVana mir are chemically modified dsRNA molecules that can mimic endogenous mir and control non-targeting miRNA is a random sequence miRNA mimic molecule that has been extensively tested in human cell lines and tissues and validated to not produce identifiable effects on known miRNA function. On the first day post-transfection, culture media was removed and replenished with new growth medium. The cells were used for in vitro experiments 72 h post transfection. For targeting each mRNA, the HSP70, BAG3 siRNA and control non-targeting siRNA was purchased from Origene (Rockville, MD, USA). For targeting each mRNA, a mixture of two or three types of siRNA duplex was used (Table S3). siRNA was transfected as described previously^57^. Briefly, cells were pre-cultured for 1 day or until 60–80% confluency and then transfected with siRNA at a final concentration of 20 nM using Lipofectamine RNAi MAX (Thermo Fisher, Waltham, MA, USA). The medium was replaced with serum-free one at 24 h post-transfection. Cells were cultured for 72 h before cell proliferation, migration and wound healing assay.

### Real-time qPCR

Total RNA preparation and RT-qPCR was carried out as described previously^58,59^. The miRNeasy mini kit (Qiagen, Hilden, Germany) was used with DNase (Qiagen). The total RNA concentration was measured by using a micro spectrophotometer Nanodrop one (Thermo Fisher, Waltham, MA, USA). cDNA synthesis was carried out by using iScriptTM cDNA Synthesis Kit (Bio-Rad, Richmond, CA. USA). The primers for reverse transcriptase are a blend of oligo (dT) and random primers. Specific primer pairs for *HSP90AA1*, *HSPA1A*, *HSPA1B*, *HSPA5*, *BAG1*, *BAG3*, *BAG4*, *CDC37*, *18s*, *TMEM11* (Table S4) were used for real-time PCR with an Applied Biosystems PowerUp SYBR Green Master mix (Thermo Fisher, Waltham, MA, USA). Relative mRNA levels to *18s* or *TMEM11* mRNA levels were quantified by the ∆∆Ct method using the formula-fold change = 2 − ∆∆Ct. PCR reaction was carried out in triplicate and mean values were calculated with the mean ± S.D. of the biological triplicates presented.

### Western blot analysis

Western Blotting was performed according to the *Cold Spring Harbor* protocol. Briefly, cells were lysed in a RIPA buffer (Boston BioProducts, MA, USA) using 25-gauge syringes. The same protein amounts were subjected to 4–20% gradient gel (Genscript, NJ, USA), followed by transfer to a polyvinylidene fluoride (PVDF) membrane using wet methods where appropriate. The membranes were blocked in blocking solution (LI-COR, Inc., Lincoln, NE, USA) for 60 min unless otherwise specified, and incubated overnight with a mouse monoclonal anti-HSP70 antibody (1/1000, SMC100, StressMarq Biosciences, BC, Canada), a rabbit polyclonal anti-BAG3 antibody (1/1000, 10599-1-AP, Proteintech, IL, USA), a mouse monoclonal anti-HSP90α antibody (1/1000, SMC147, StressMarq, Biosciences, BC, Canada) and a rabbit polyclonal anti-GAPDH antibody (1/1000, sc-25778, Santa Cruz, CA, USA). The membranes were incubated for 1 h at room temperature with goat anti-rabbit IRDye 800 CW fluorescent secondary antibodies (1/15,000, LI-COR, Inc., Lincoln, NE, USA) and 680 RD fluorescent secondary antibodies (1/15,000, LI-COR, Inc., Lincoln, NE, USA). Blots were washed with Tris buffered saline, 0.1% (w/v) Tween 20 (TBS-T) and visualized with the Odyssey Imaging System (LI-COR, Inc., Lincoln, NE, USA). The quantitative densitometric analysis was performed using Image Studio Lite Ver. 5.2 (LI-COR, Inc., Lincoln, NE, USA).

### Total protein staining

Protein samples (5 μg each) were loaded on 4–20% gradient gel (Genscript, NJ, USA). After the electrophoresis run, the gel transferred to PVDF membrane using wet methods. The membrane was hydrated using 100% methanol for 30 s and washed with tris-buffered saline, pH7.4 (TBS) for 5 min at room temperature with gentle shaking. The membrane was stained with Revert 700 total protein stain solution (LI-COR, Inc., Lincoln, NE, USA) and incubated for 5 min at room temperature with washing solution (LI-COR, Inc., Lincoln, NE, USA). The membrane was then washed with Revert 700 Wash Solution n (LI-COR, Inc., Lincoln, NE, USA), rinsed two times for 30 s at room temperature with gentle shaking. The membrane was then briefly rinse with ultrapure water. For visualizing the membrane, the membrane was imaged in the 700 nm channel using Image Studio Lite Ver. 5.2 (LI-COR, Inc., Lincoln, NE, USA).

### Heat shock/thermotolerance conditions

Control non-targeting miRNA and mir-570 (25 or 50 nM) transfected HEK293T cells were either untreated (37 °C) or treated with heat shock at 43 °C for 30 min. For thermotolerance experiment, cells were treated with heat shock recovered at 37 °C for 6 h. Cells were then treated with a second heat shock at 43 °C for 90 min followed by 37 °C recovery for 6 h. Cells were seeded in 96-well plate for the cell proliferation assay and 6 cm dish for the colony formation assay.

### Detection of colony formation by crystal violet

Cells (1.0 × 10^3^ cells/plate) suspended in DMEM medium with 10% FBS were added to 6 cm dish. Ten days post-treatment, colonies were visualized by crystal violet staining. The medium was replaced every 3 days during the analysis of colony formation. The supernatants were discarded and the remaining viable adherent cells were fixed with paraformaldehyde for 10 min then stained with 0.5% crystal violet in 25% methanol for 10 min. Cells were then rinsed with water and allow to dry overnight. Percentage of colonies formed were the averages calculated from three individual plates.

### Hsp90 inhibitor treatment

Assay of cell proliferation with and without Hsp90 inhibitor (17-AAG) in cells treated with miRNA-570. After treatment with mir-570 or control miRNA, the proliferation of SKBR3 cells was evaluated with and without 17-AAG treatment. Cells were counted by brightfield imaging cytometry at time 0 and after 72 h. Dose–response curves with IC50 values for 17-AAG in respect to treatment to mir-570 and control non-targeting miRNA. The results are expressed as percentage of absorbance measurement after 3-(4,5-Dimethylthiazol-2-yl)-2,5-diphenyl tetrazoliumbromide (MTT) assay^60^. The calculated regression curve is indicated as a line. MTT was used to assay mitochondrial activity in viable cells. Cells were plated at a concentration of 1.0 × 10^4^ cells/well in 96-well tissue culture plates in a medium containing 10% FBS and cultured for 24 h.

### Cell proliferation assay

Cell proliferation assay was carried out as described previously^61^. For analysis of cell proliferation, cells were seeded at a concentration of 2.0 × 10^3^ cells/well in a 96-well plate. The number of cells at 1 to 7 days post-seeding period was counted by using Hemocytometer (LW scientific, GA, USA). The medium was replaced with fresh ones every 3 days during the analysis of cell proliferation.

### Cell migration assay

Multiwell cell migration assays were performed following established protocol^62,63^. Multiwell chambers were incubated for the indicated time upon which SKBR3 cells on the upper side of filter were removed and those on the underside were then fixed and stained with 0.5% crystal violet. Migrated cells were counted using 20 × magnification.

### Wound healing assay

Confluent cells in 96-well plates were “wounded” by scratching a line in the monolayer with a sterile Pasteur pipette tip. Images were captured immediately and subsequently at 24 h using a Floid® Imaging Station. The percentage of the area closed in 24 h was measured using Image J.

### Statistical analysis

Statistical significance was calculated using JMP Pro 15 and Microsoft Excel. Three or more mean values were compared using one-way analysis of variance (ANOVA) followed by post hoc Turkey’s test, while comparisons of 2 were done with an unpaired Student's t-test. *p < 0.05, **p < 0.01, ***p < 0.001, ****p < 0.0001 was considered to indicate statistical significance.

## Supplementary Information

Supplementary Information.

## Data Availability

The datasets used and/or analyzed during the current study are available from the corresponding authors on reasonable request.

## Abbreviations

Mir: microRNA
HSP: Heat shock protein
BAG3: BAG family molecular chaperone regulator 3
CCT: Chaperonin containing tailless complex polypeptide 1
MMP: Matrix metalloproteinase
MIR-570: ds-miR-570
MIR-NT: ds-miR-non targeting
HS: Heat shock
MMT: 3-(4,5-Dimethylthiazol-2-yl)-2,5-diphenyl tetrazoliumbromide
