# Supplementary material for: MicroRNA-570 targets the HSP chaperone network, increases proteotoxic stress and inhibits mammary tumor cell migration
Source: Sci Rep. 2022 Sep 16;12:15582. doi: 10.1038/s41598-022-19533-6 (PMC9481609; doi:10.1038/s41598-022-19533-6)
Supplement: Supplementary file 1 — Supplementary Information. [file 41598_2022_19533_MOESM1_ESM.pdf]

Supplementary information

MicroRNA-570 targets the HSP chaperone network, increases proteotoxic stress and inhibits mammary tumor cell migration

Yuka Okusha, Martin E. Guerrero-Gimenez, Benjamin J. Lang, Thiago J. Borges, Mary A. Stevenson, Andrew W. Truman and Stuart K. Calderwood

Table S1. Co-expression correlation HSPs and co-chaperones in patient-derived breast tumor samples.
Table S2. Targeting genes of three candidate miRNAs in chaperome, co-chaperones and ECM related genes.
Table S3. The sequence of siRNA.
Table S4. The sequence of primers for RT-qPCR.
Figure S1. Scheme of binding of miRNAs to *HSPA1A, HSP90AA1* and *BAG3* mRNA.
Figure S2. Characterization of *HSPA5, BAG1, BAG4* and *CDC37* as novel direct targets of mir-570.
Figure S3. The expression of HSP90β, HSC70 and BAG2 by mir-570 transfection.
Figure S4. Activation of the heat shock response leads to the synthesis of HSPs and the suppressive effects of mir-570 on HSP and BAG3 expression.
Figure S5. Full images of western blotting of HSP90α (A), HSP90β(B), HSP70 (C), HSC70 (D), BAG2 (E), BAG3 and GAPDH (F), supporting Fig. 1C.
Figure S6. Full images of western blotting of HSP70 (A), BAG3 (B), HSP90α (C) and GAPDH (D), supporting Fig. 4B.
Figure S7. Full images of western blotting of HSP90α (A), HSP70 (B), BAG3 (C) and GAPDH (D), supporting Fig. 5A.
Figure S8. Full images of western blotting of HSP90α (A), HSP70 (B), BAG3 (C) and GAPDH (D), supporting Fig. 6B.
Figure S9. Full images of western blotting of HSP90α (A), HSP70 (B), BAG3 (C) and GAPDH (D), supporting Fig. 6C.
Figure S10. Full images of western blotting of HSP70 (A), BAG3 (B) and GAPDH (C), supporting Fig. 8A.
Figure S11. Full images of western blotting of HSP90β (A), HSC70 (B), BAG2 (C) and GAPDH (D), supporting Fig. S3.
Figure S12. Full images of western blotting of HSP90α (A), HSP70 (B), BAG3 (C) and GAPDH (D), supporting Fig. S4A.
Figure S13. Full images of western blotting of HSP90α (A), HSP70 (B), BAG3 (C) and GAPDH (D), supporting Fig. S4B.
Figure S14. Full images of western blotting of HSP90α (A), HSP70 (B), BAG3 (C) and GAPDH (D), supporting Fig. S4C.

Table S1. Co-expression correlation HSPs and co-chaperones in patient-derived breast tumor samples.

Gene	Correlated Gene	Cytoband	Spearman's Correlation	p-Value	q-Value
HSPA1A	HSPA1B	6p21.33	0.846	2.37E-264	4.74E-260
HSPA1A	BAG3	10q26.11	0.328	1.83E-25	9.20E-24
HSPA1A	HSP90AA1	14q32.31	0.217	1.12E-11	7.92E-11
HSPA1A	HSP90AB1	6p21.1	0.343	7.93E-28	5.53E-26
HSPA1B	BAG3	10q26.11	0.24	4.76E-14	6.41E-13
HSPA1B	HSP90AA1	14q32.31	0.271	1.15E-17	3.09E-16
HSPA1B	HSP90AB1	6p21.1	0.437	5.79E-46	2.90E-42
HSP90AB1	HSP90AA1	14q32.31	0.55	6.06E-77	1.21E-73
HSP90AB1	HSPA8	11q24.1	0.386	1.58E-35	6.40E-34
HSPA8	HSP90AA1	14q32.31	0.463	3.28E-52	1.60E-49
BAG3	BAG1	9p13.3	0.142	9.55E-06	4.37E-05

Table S2 . Targeting genes of three candidate miRNAs in chaperome, co-chaperones and ECM related genes.

Category	Mir570	Mir224	Mir522
HSP70/HSP90	**HSPA1A**<br>**HSPA1B**<br>**HSP90AA1**<br>HSPA5	**HSPA1A**<br>HSPA8<br>HSP90B1<br>HSPH1<br>HSPA14	**HSPA1A**<br>HSPA8<br>HSP90B1<br>HSPH1<br>HSPA14
Co-chaperones	**BAG3**<br>BAG1<br>BAG4<br>**CDC37**	**BAG3**<br>BAG1	**BAG3**<br>BAG1
DNAJ	DNAJB4<br>DNAJB6<br>DNAJC10<br>DNAJA2<br>DNAJA3<br>DNAJA4<br>DNAJB1<br>DNAJC7	DNAJB4<br>DNAJB6<br>DNAJC10	DNAJB4<br>DNAJB6<br>DNAJC10<br>DNAJA1<br>DNAJC24
CCT	CCT5<br>CCT8		CCT3
MMPs	MMP24<br>MMP1<br>MMP11	MMP24<br>MMP15<br>MMP16<br>MMP19	MMP24<br>MMP15<br>MMP16<br>MMP19
Collagen	Col5A2<br>Col11A1<br>Col14A1<br>Col8A2<br>Col5A1<br>Col15A1<br>ColQ	Col5A2<br>Col11A1<br>Col14A1<br>Col1A1<br>Col1A2<br>Col4A1<br>Col4A2<br>Col4A3<br>Col4A4<br>Col4A6<br>Col8A1	Col5A2<br>Col11A1<br>Col14A1<br>Col1A1<br>Col1A2<br>Col4A1<br>Col4A2<br>Col4A3<br>Col4A4<br>Col4A6

Table S3. The sequence of siRNA.

Name of siRNA	Sequence (5' to 3')
hHSPA1A	GAUGAAUUUAUACUGCCAUCUUACGCG<br>GACUUUGCAUUUCCUAGUAUUUCTGTG<br>UUCAAGACUUUGCAUUUCCUAGUATAT
hHSPA1B	CCGAUAUGUUCAUUAGAAUUCUUTGTG<br>ACACUACAAAGGCUGGGAAUGUATGTG<br>AGUUGUAACCUGAUGGUAAUUAGCTCT
hBAG3	GCUGUAGACAACUUUGAAGGCAAGAGA<br>GCCAUAGGAAUAUCUGUAUGUUGGAGA<br>CCUGAUGAUCGAAGAGUAUUUGACCCC

Table S4. The sequence of primers for RT-qPCR.

Name of primers	Sequence (5' to 3')
h HSP90AA1 Fw	CATCTCCATGATTGGGCAGTT
h HSP90AA1 Rv	CTTTGACCCGCCTCTCTTCTA
h HSPA1A Fw	GCCTTTCCAAGATTGCTGTT
h HSPA1A Rv	TCAACATTGCAAACACAGGA
h HSPA1B Fw	AGGGTGTTTCGTTCCCTTTA
h HSPA1B Rv	CATTCCCAGCCTTTGTAGTG
h HSPA5 Fw	TTGGAGGTGGGCAAACAAAG
h HSPA5 Rv	TCTTTGGTTGCTTGGCGTTG
h BAG1 Fw	AACACCGTTGTCAGCACTTG
h BAG1 Rv	TCAGCTTGCAAATCCTTGGG
h BAG3 Fw	TGGGAGATCAAGATGGACCC
h BAG3 Rv	GGGCCATTGGCAGAGGATG
h BAG4 Fw	TACGACCACAAGAAGATGCG
h BAG4 Rv	TTGTGCCGGTTCATGCTTTG
h CDC37 Fw	AGAAGAGCATGCCCTGGAAC
h CDC37 Rv	GTTTCTGCTCCCTCACCTCC
h 18s Fw	AACCCGTTGAACCCCATT
h 18s Rv	CCATCCAATCGGTAGTAGCG
h TMEM11 Fw	TCCTGGCAGTTTGACCCTTGCT
h TMEM11 Rv	CAGTCTCTTTCTGTGCAGGTCG

A

```
HSPA1A 5′...AAGAUUGCUGUUUUUGUUUUGG... 3′     HSP90AA1 5′...CUCUCCACAGGGCUUGUUUUCC... 3′
                         ||||||                                         |||||||
Mir570 3′  CGUUUCCAUUAACGACAAAAGC    5′     Mir570   3′  CGUUUCCAUUAACGACAAAAGC    5′
```

B

```
HSPA1A 5′...UCUGGGUCAGGCCCCACCAUUGA...3′     BAG3     5′...UUUAUCAGAAAUGUUGCCAUUUU...3′
                         |||||||                                          ||||||
Mir224 3′  ACAUCAGUGAUCCCGUGGUAAAA 5′     Mir224   3′  ACAUCAGUGAUCCCGUGGUAAAA 5′
```

C

```
HSPA1A 5′...CUGGGUCAGGCCCCACCAUUGA... 3′     BAG3     5′...UUAUCAGAAAUGUUGCCAUUUU... 3′
                         ||||||                                         ||||||
Mir522 3′  UGUGAGAUUUCCCUUGGUAAAA    5′     Mir522   3′  UGUGAGAUUUCCCUUGGUAAAA    5′
```

Figure S1. Scheme of binding of miRNAs to *HSPA1A, HSP90AA1* and *BAG3* mRNA.
(A) Scheme of binding of mir-570 to *HSPA1A* and *HSP90AA1* mRNA.
(B) Scheme of binding of mir-224 to *HSPA1A* and *BAG3* mRNA.
(C) Scheme of binding of mir-522 to *HSPA1A* and *BAG3* mRNA. The figure shows sequences from the 3'UTR regions of the target genes and the microRNA predicted to bind to them according to the 'miRanda' algorithm.

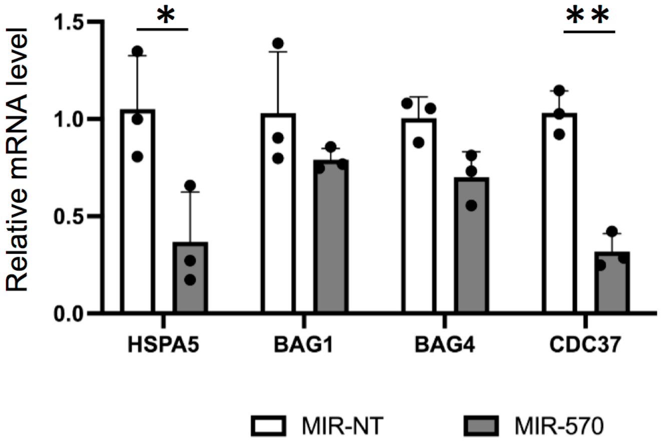

Figure S2. Characterization of *HSPA5, BAG1, BAG4* and *CDC37* as novel direct targets of mir-570. Real-time qPCR analysis of *HSPA5, BAG1, BAG4 and CDC37* in HEK293T cells 72 h after transfection with 50 nM of dsRNA mimicking miR-570 (MIR-570) or control non-specific miRNA (MIR-NT). Representative image of three individual experiments with three replicates (n=3; *p<0.05, * * p<0.01, Student's t-test, error bars = SD).

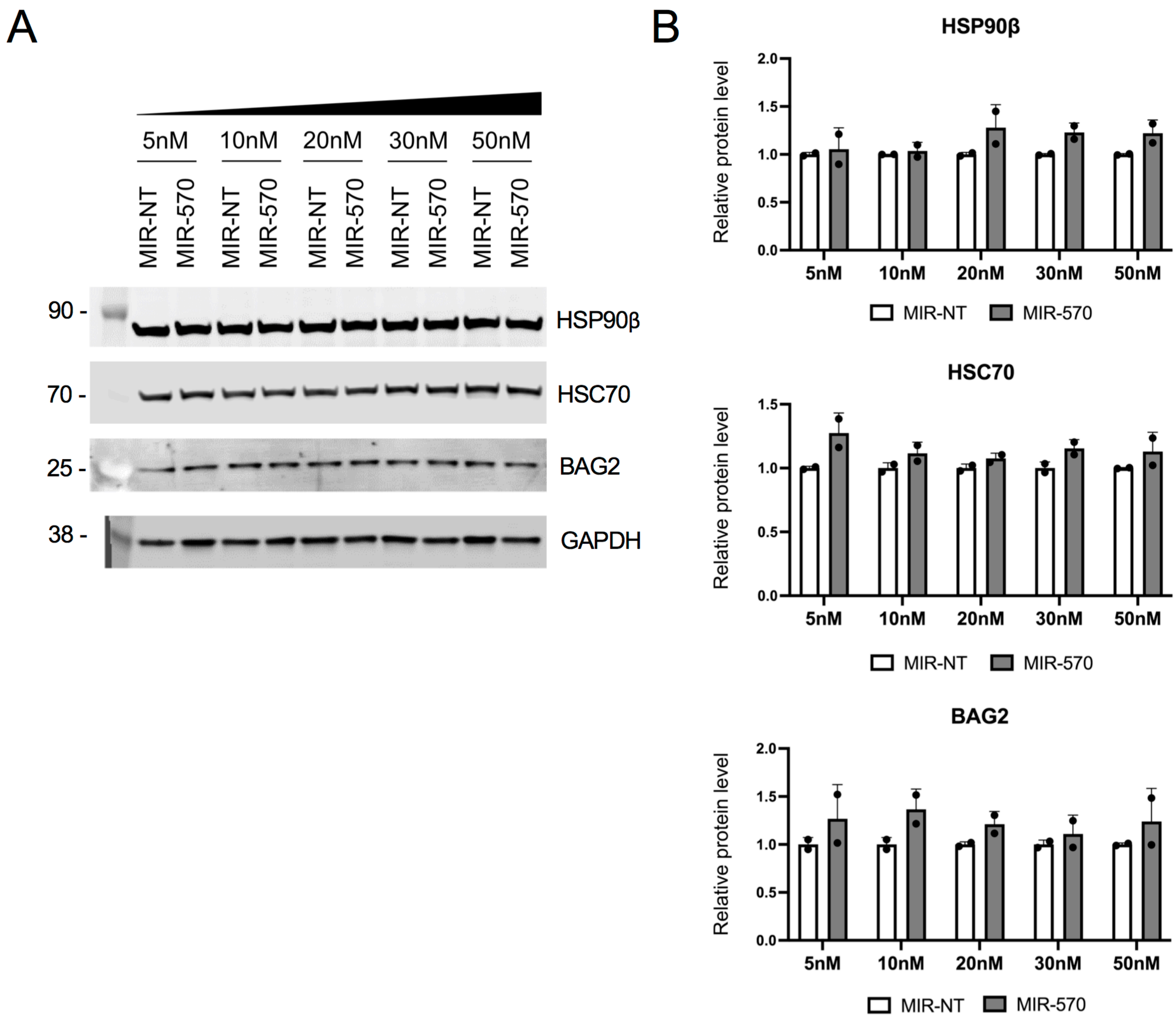

Figure S3. The expression of HSP90β, HSC70 and BAG2 by mir-570 transfection.
(A) Western blot analysis of HSP90β, HSC70, BAG2 and GAPDH in HEK293T cells 72 h after transfection with a range of concentrations, 5 nM to 50 nM, of mir-570 or MIR-NT. Molecular weight markers are indicated in kilodalton. Original blots are presented in Supplementary Figure 11.
(B) Quantitative analyses of western blot for HSP90β, HSC70 and BAG2 of two individual experiments. Individual data points are indicated by open circles. GAPDH was used as an internal control.

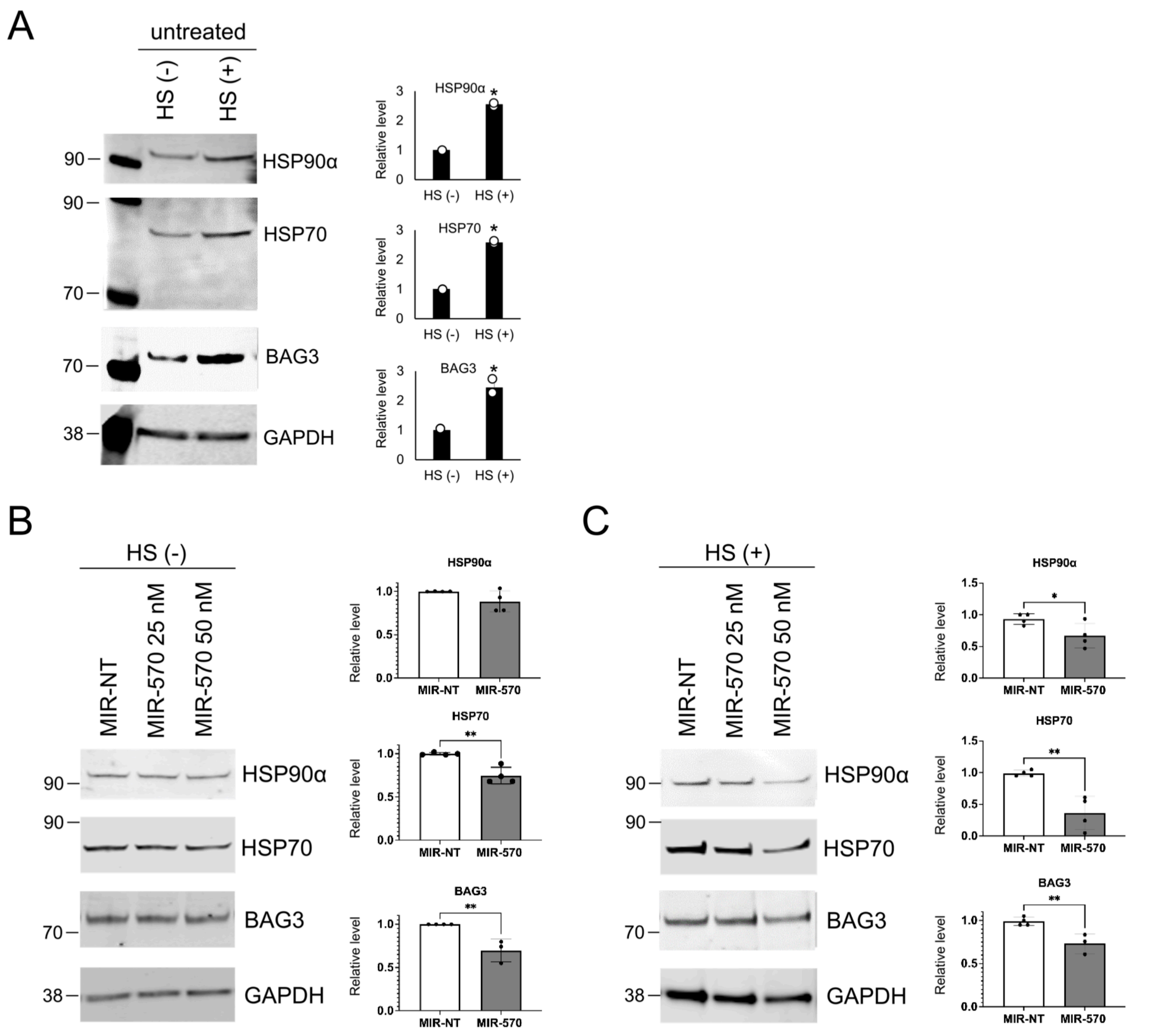

Figure S4. Activation of the heat shock response leads to the synthesis of HSPs and the suppressive effects of mir-570 on HSP and BAG3 expression.

(A) Western blot analysis of HSP90α, HSP70 and BAG3 in HEK293T cells 6h after heat shock treatment. Quantitative analyses of western blot for HSP70, BAG3 and HSP90α. Levels of GAPDH expression were examined as loading control. Molecular weight markers are indicated in kilodalton. The data shows mean ± SD obtained in the biological triplicate assay and the individual data points were indicated in a circle (n=3; *p<0.05, Student's t-test, error bars = SD). Original blots are presented in Supplementary Figure 12.

(B) Left: western blot analysis of HSP90α, HSP70 and BAG3 in HEK293T cells 72 h after transfection with 25 nM or 50 nM of MIR-570 or 50 nM of MIR-NT with no exposure to heat shock. Right: quantitative analyses of western blot for HSP90α, HSP70 and BAG3 by mir-570 transfection (50 nM) of three or four individual experiments and the individual data points were indicated in a circle. (*p<0.05, * * p<0.05, Student's t-test, error bars = SD). Levels of GAPDH expression were examined as loading control. Molecular weight markers are indicated in kilodalton. Original blots are presented in Supplementary Figure 13.

(C) Left: western blot analysis of HSP90α, HSP70 and BAG3 in HEK293T cells 72 h after transfection with 25 nM or 50 nM of MIR-570 or 50 nM of MIR-NT and with exposure to heat shock. Right: quantitative analyses of western blot for HSP90α, HSP70 and BAG3 by mir-570 transfection (50 nM) of three or four individual experiments and the individual data points were indicated in a circle. (*p<0.05, * * p<0.05, Student's t-test, error bars = SD). Levels of GAPDH expression were examined as loading control. Molecular weight markers are indicated in kilodalton. Original blots are presented in Supplementary Figure 14.

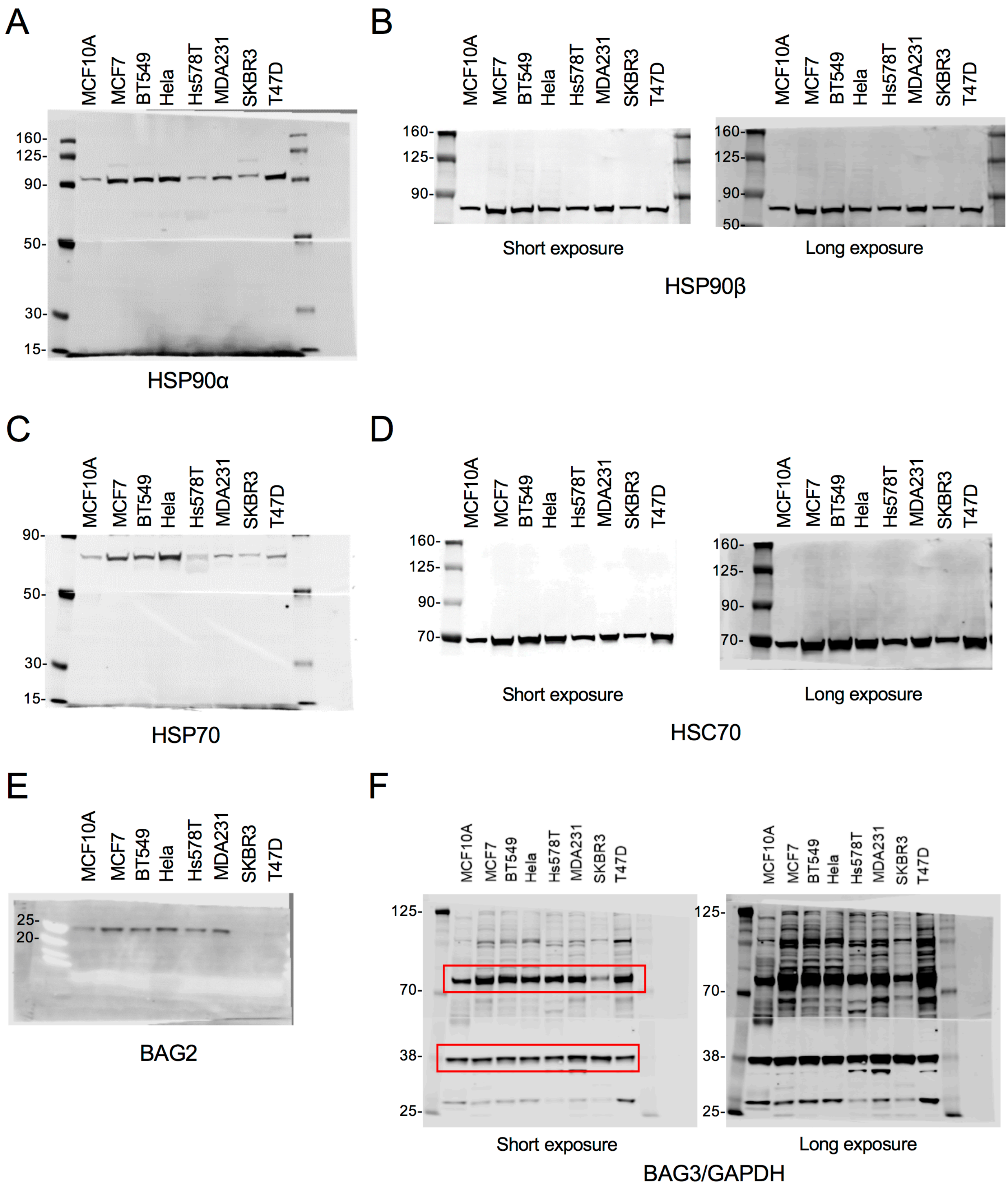

Figure S5. Full images of western blotting of HSP90α (A), HSP90β(B), HSP70 (C), HSC70 (D), BAG2 (E), BAG3 and GAPDH (F), supporting Fig. 1C.

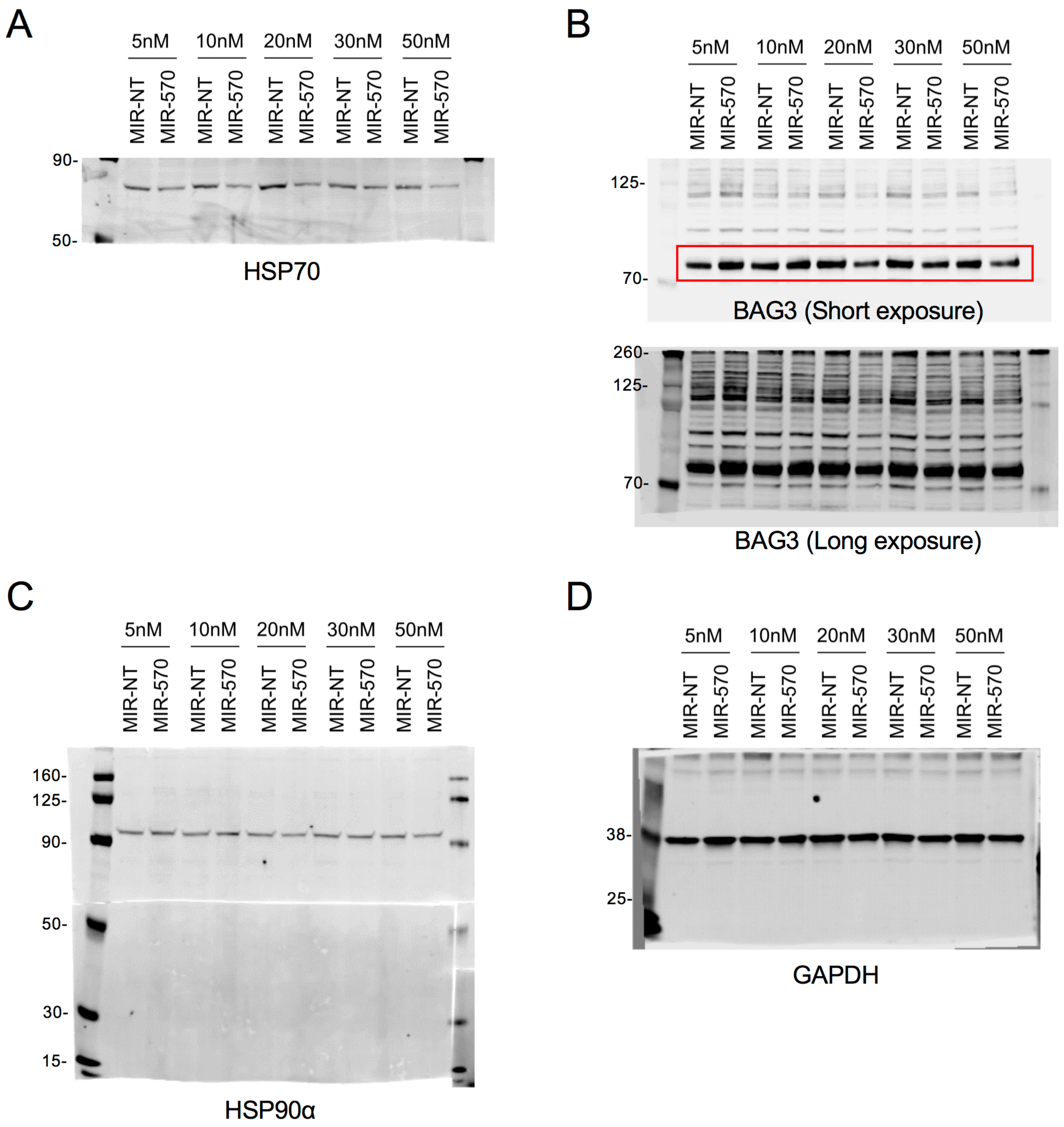

Figure S6. Full images of western blotting of HSP70 (A), BAG3 (B), HSP90α (C) and GAPDH (D), supporting Fig. 4B.

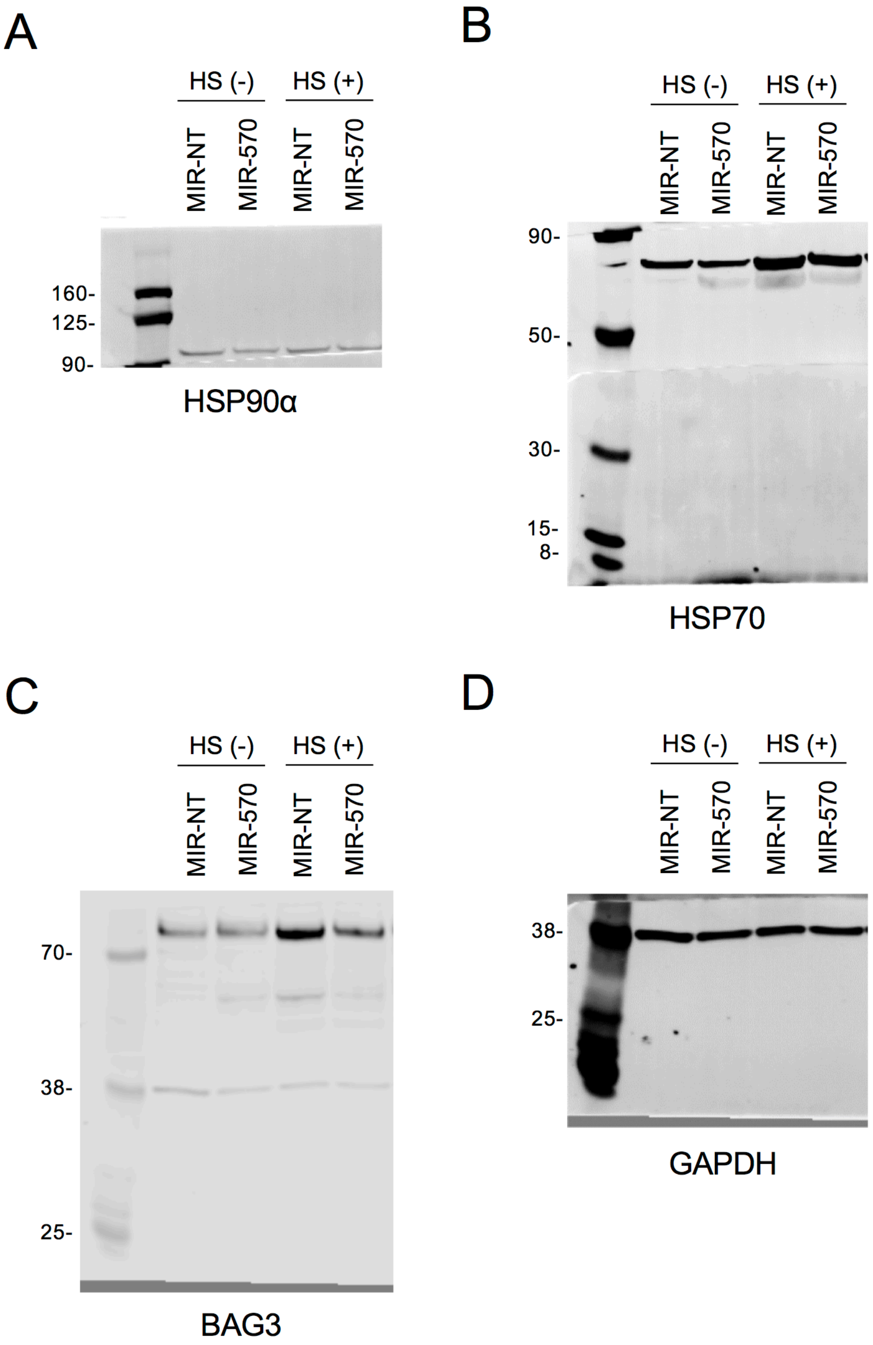

Figure S7. Full images of western blotting of HSP90α (A), HSP70 (B), BAG3 (C) and GAPDH (D), supporting Fig. 5A.

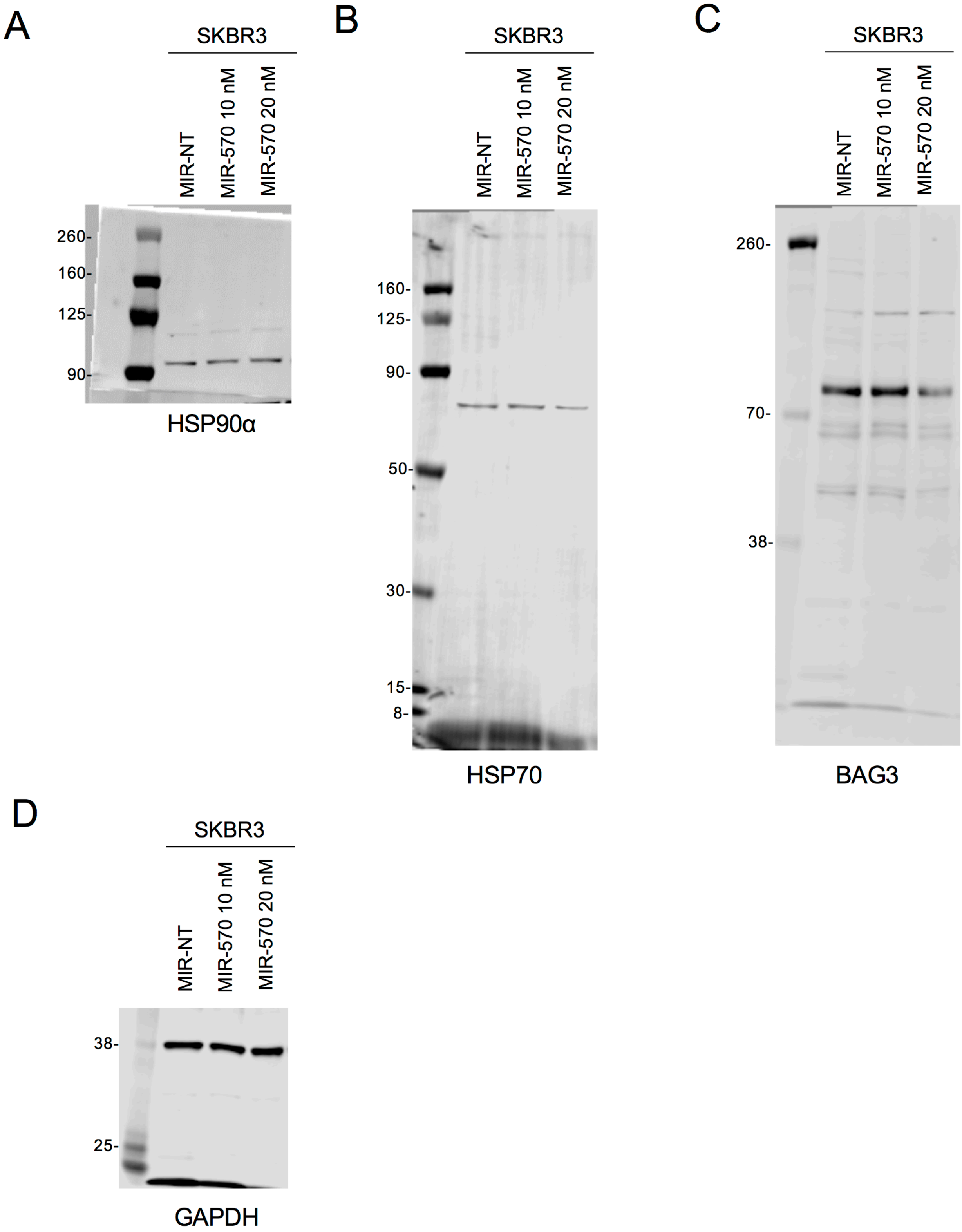

Figure S8. Full images of western blotting of HSP90α (A), HSP70 (B), BAG3 (C) and GAPDH (D), supporting Fig. 6B.

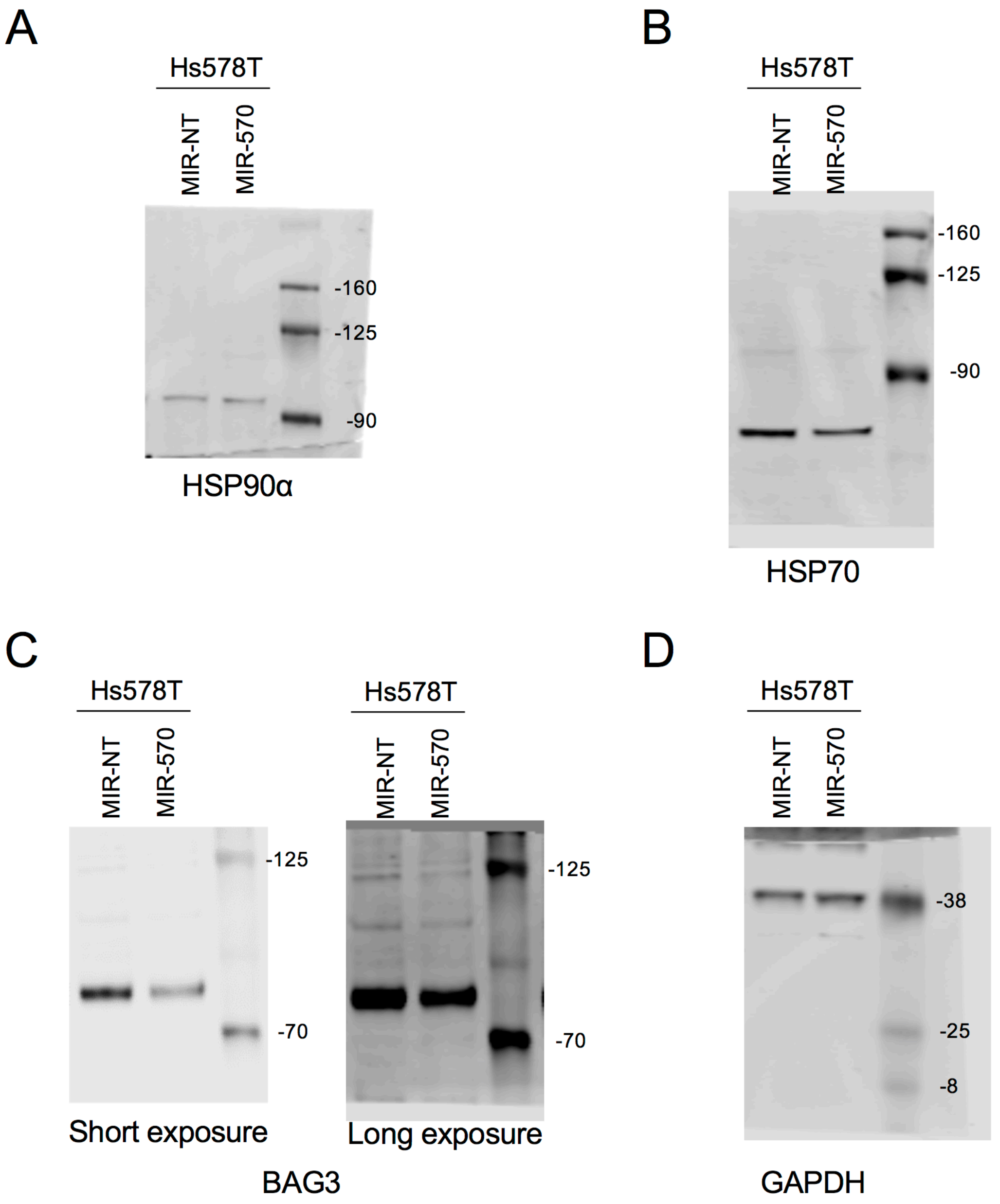

Figure S9. Full images of western blotting of HSP90α (A), HSP70 (B), BAG3 (C) and GAPDH (D), supporting Fig. 6C.

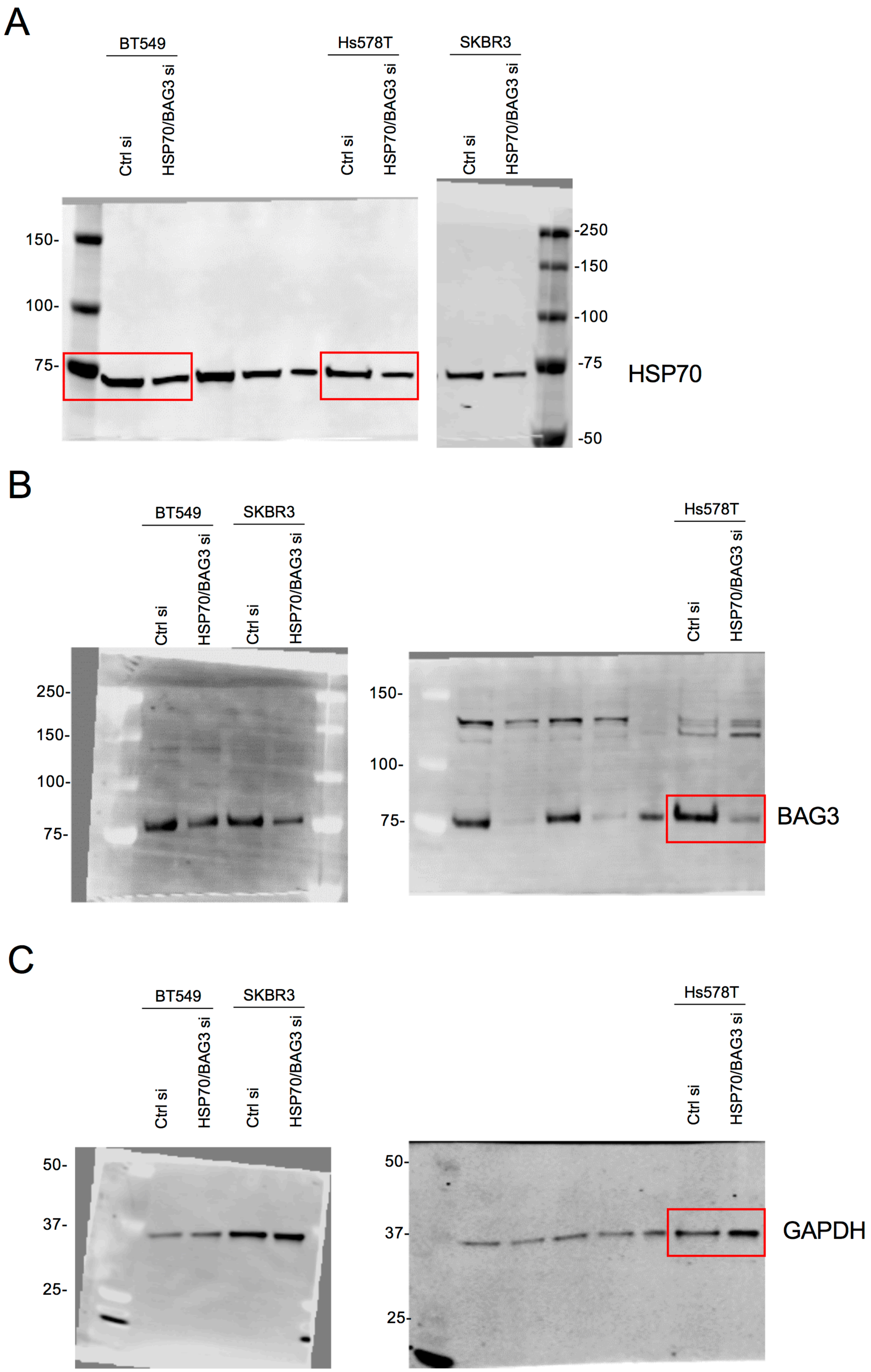

Figure S10. Full images of western blotting of HSP70 (A), BAG3 (B) and GAPDH (C), supporting Fig. 8A.

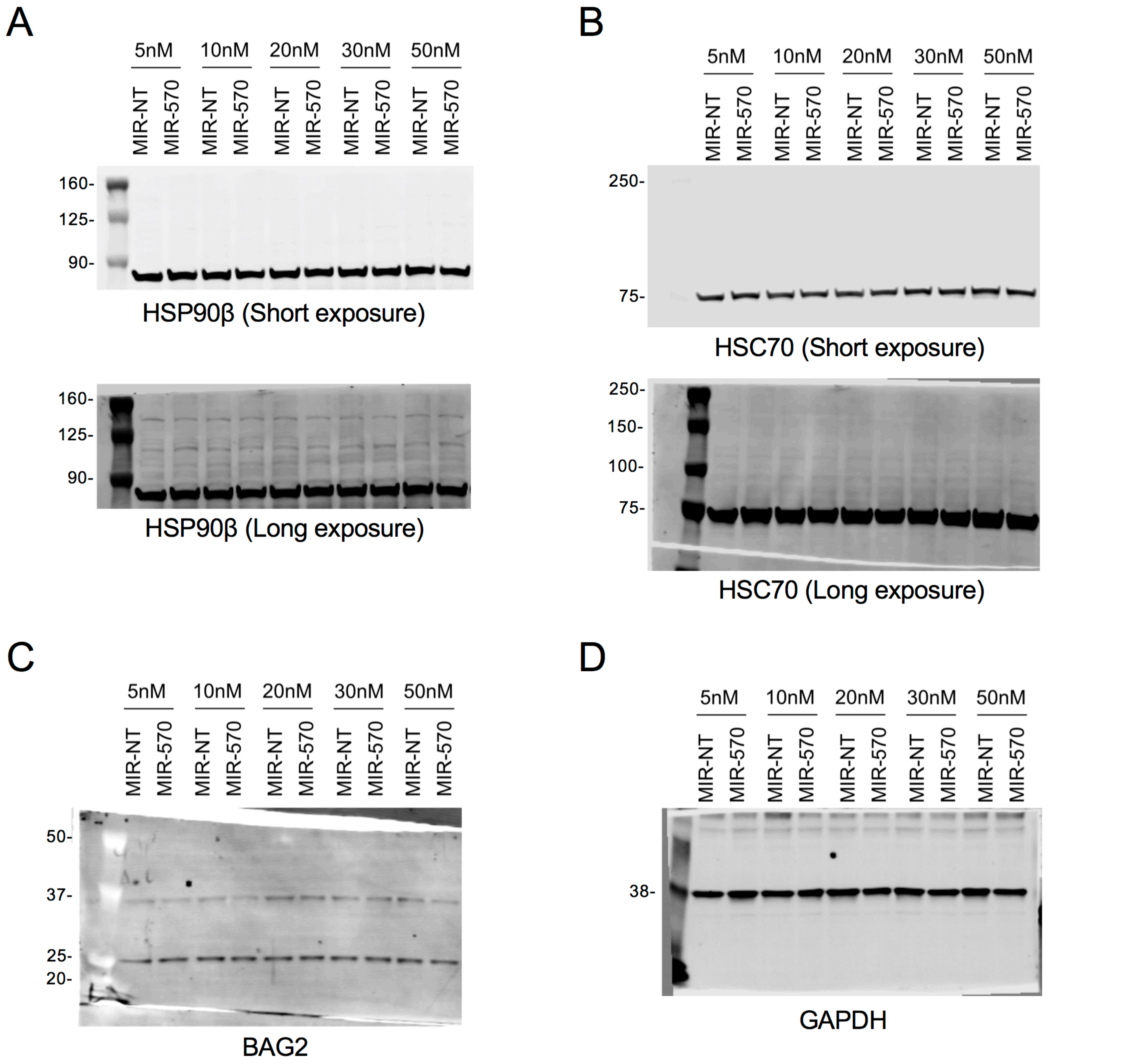

Figure S11. Full images of western blotting of HSP90β (A), HSC70 (B), BAG2 (C) and GAPDH (D), supporting Fig. S3.

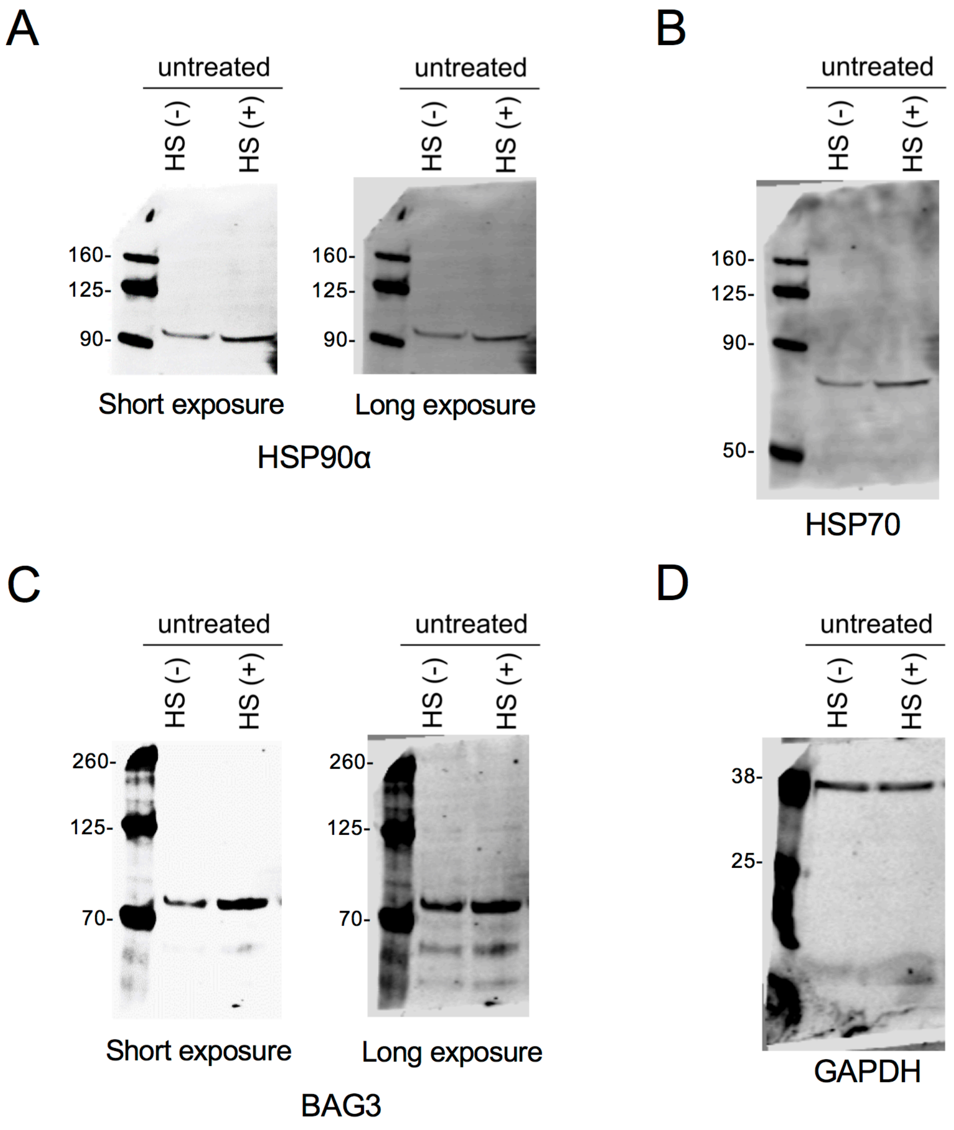

Figure S12. Full images of western blotting of HSP90α (A), HSP70 (B), BAG3 (C) and GAPDH (D), supporting Fig. S4A.

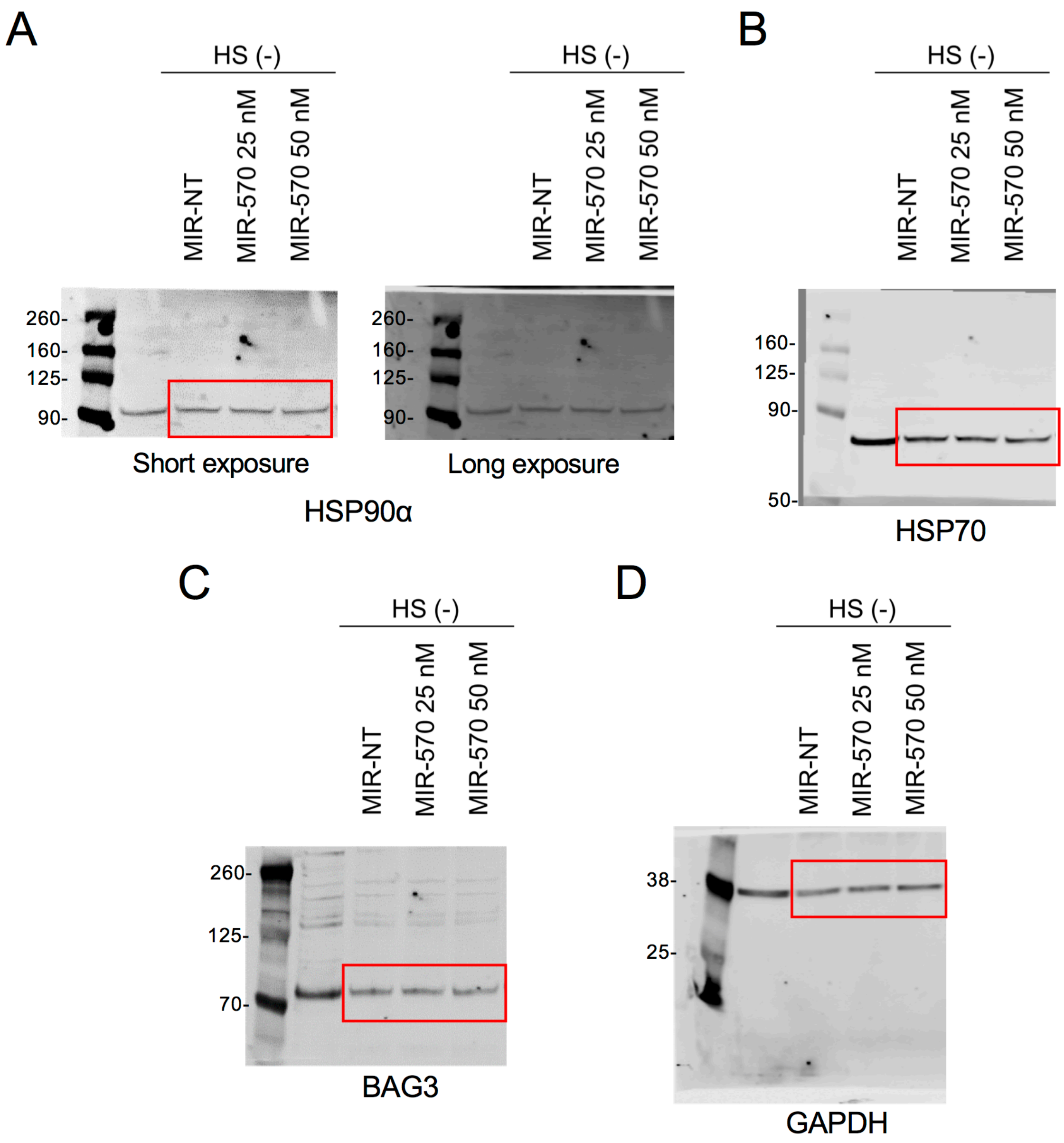

Figure S13. Full images of western blotting of HSP90α (A), HSP70 (B), BAG3 (C) and GAPDH (D), supporting Fig. S4B.

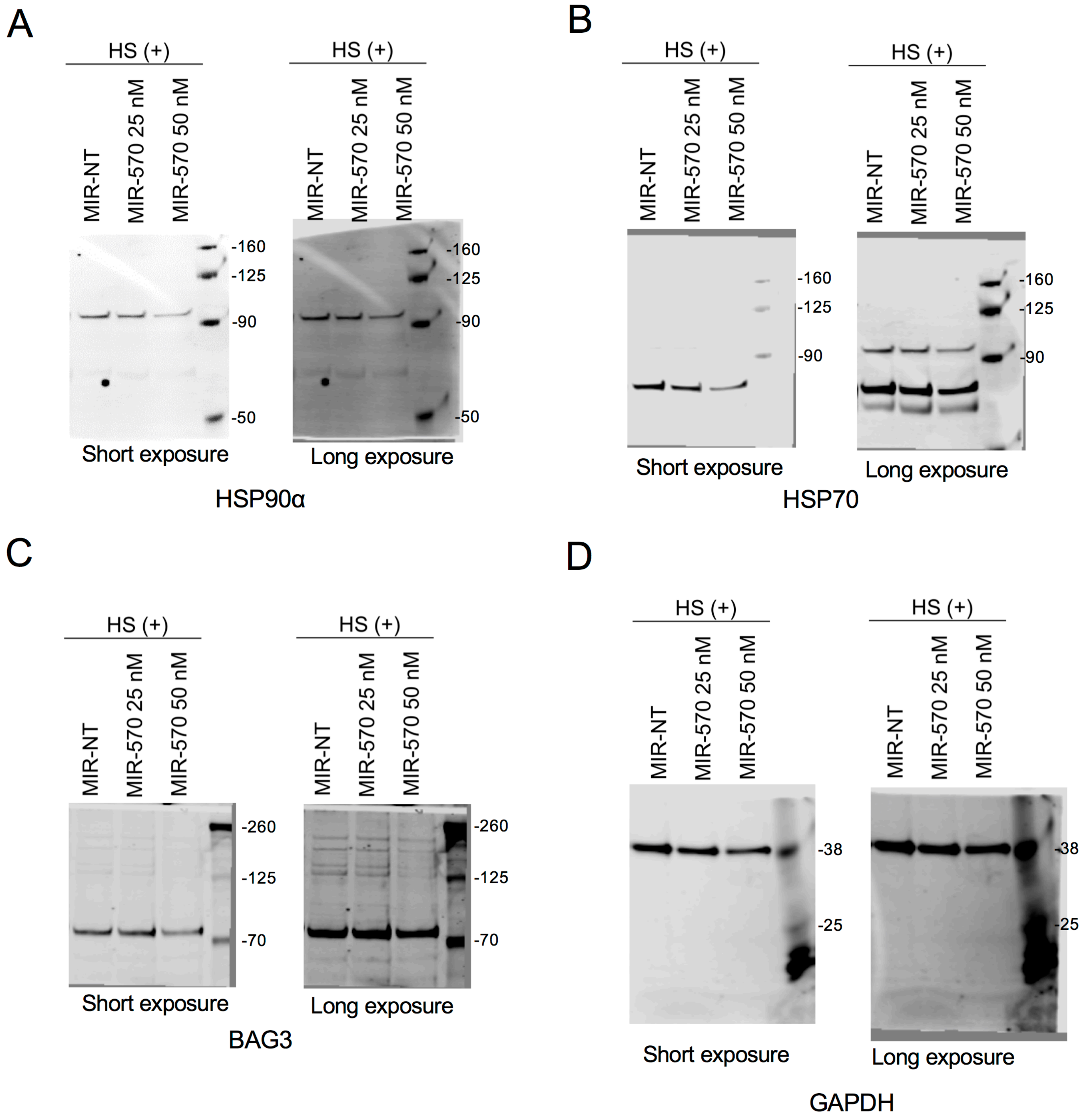

Figure S14. Full images of western blotting of HSP90α (A), HSP70 (B), BAG3 (C) and GAPDH (D), supporting Fig. S4C.
